# Valorization of Brewery Wastes for the Synthesis of Silver Nanocomposites Containing Orthophosphate

**DOI:** 10.3390/nano11102659

**Published:** 2021-10-10

**Authors:** Alcina Johnson Sudagar, Neha Venkatesh Rangam, Artur Ruszczak, Paweł Borowicz, József Tóth, László Kövér, Dorota Michałowska, Marek Ł. Roszko, Krzysztof R. Noworyta, Beata Lesiak

**Affiliations:** 1 Institute of Physical Chemistry, Polish Academy of Sciences, Kasprzaka 44/52, 01-224 Warsaw, Poland; aruszczak@ichf.edu.pl (A.R.); pborowicz@ichf.edu.pl (P.B.); knoworyta@ichf.edu.pl (K.R.N.); blesiak-orlowska@ichf.edu.pl (B.L.); 2 Institute for Nuclear Research, BemTér 18/c, H-4026 Debrecen, Hungary; toth.jozsef@atomki.hu (J.T.); kover.laszlo@atomki.hu (L.K.); 3 Institute of Agriculture and Food Biotechnology—State Research Institute, ul. Rakowiecka 36, 02-532 Warsaw, Poland; dorota.michalowska@ibprs.pl (D.M.); marek.roszko@ibprs.pl (M.Ł.R.)

**Keywords:** green chemistry, nanoparticles, waste valorization, antibacterial, silver phosphate

## Abstract

Brewery wastes from stage 5 (Wort precipitate: BW5) and stage 7 (Brewer’s spent yeast: BW7) were valorized for the synthesis of silver phosphate nanocomposites. Nanoparticles were synthesized by converting silver salt in the presence of brewery wastes at different temperatures (25, 50, and 80 °C) and times (10, 30, and 120 min). Unexpectedly, BW7 yielded Ag_3_PO_4_ nanoparticles with minor contents of AgCl and Ag metal (Ag_met_). Contrastingly, BW5 produced AgCl nanoparticles with minor amounts of Ag_3_PO_4_ and Ag_met_. Nanocomposites with different component ratios were obtained by simply varying the synthesis temperature and time. The morphology of the nanocomposites contained ball-like structures representative of Ag_3_PO_4_ and stacked layers and fused particles representing AgCl and Ag_met_. The capping on the nanoparticles contained organic groups from the brewery by-products, and the surface overlayer had a rich chemical composition. The organic overlayers on BW7 nanocomposites were thinner than those on BW5 nanocomposites. Notably, the nanocomposites exhibited high antibacterial activity against *Escherichia coli* ATCC 25922. The antibacterial activity was higher for BW7 nanocomposites due to a larger silver phosphate content in the composition and a thin organic overlayer. The growth of Ag_met_ in the structure adversely affected the antimicrobial property of the nanocomposites.

## 1. Introduction

Globally, the brewing industry is a significant source of waste effluents. Management of this waste is increasingly a problem due to the increased annual production rate [1]. Brewery industry wastes have not been widely explored as materials for synthesis and particularly not in terms of nanoparticle synthesis. However, brewery waste analysis has been performed to determine its constituents. Amino acids, phosphates, polyphenols, proteins, and sugars have been determined and quantified [2]. Brewery waste remediation and valorization research have shown potential in different application fields. Brewery waste has been used as the carbon source for the Bacillus Subtilis N3-1P bacterium to synthesize biosurfactants [3]. Fractionation and purification of brewery waste have been performed to obtain bioactive compounds in food, pharmaceutical, agricultural, cosmetic, and chemical industries [4,5]. Nevertheless, there is still much scope in the field of brewery wastes valorization.

**Scheme 1:**
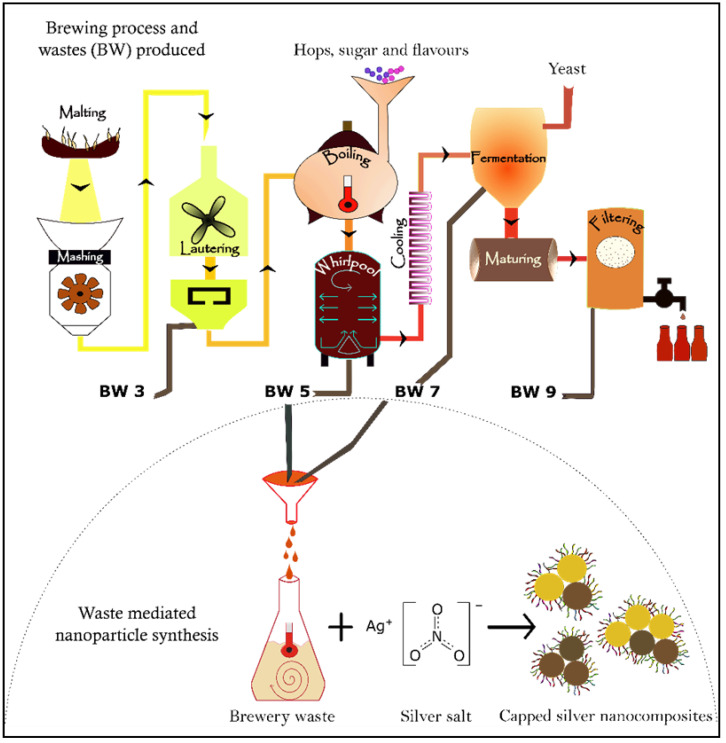
Wastes (BW) produced in brewing and their valorization for nanoparticle synthesis.

The production of malt beer occurs in a 10-step process that leads to brewery waste at various production stages (Scheme 1) [6]. Malt is a sprouted and dried cereal grain, in this case, barley. Malting comes down to steeping the cleaned grain and maintaining its moisture for a sufficiently long time. Steeping, which lasts from three to four days, aims to wake the embryos from sleep and initiate germination. This process is accompanied by the secretion of enzymes in the grain that break down excess protein and sugars. Amylase helps break down barley starch into simple sugars and disaccharides (primarily maltose). It is vital to break down as many spare materials into simple sugars and amino acids in malting. The malt is then milled, mixed with water, and mashed to dissolve the starch and protein, releasing sugar and tannin. The sweet malt extract called wort is separated from the solid substances at the third stage called Brewer’s spent grains (BSG) and is the first type of waste, BW3. In the following stage, the wort is boiled after adding the bitter, aromatic hops and sometimes sugar. The tannin and part of proteins are separated during boiling. The wort is clarified in a whirlpool and the clear wort is tapped out. The undissolved hop particles and proteins are collected at the whirlpool center, forming the second type of waste (BW5) called wort precipitate. The clear wort is then cooled and fermented by adding yeast. Absorbed nutrients not used by the embryo are the substrate of the alcoholic fermentation carried out by yeast in the brewery while brewing beer. The yeast filtered out after fermentation called Brewer’s spent yeast (BSY) at the seventh stage forms the third type of waste, BW7. After maturing, the fermented beer is filtered using diatomaceous earth before going into the production line and yielding the final product, beer B. The waste, including diatomaceous earth, separated at this ninth stage, forms the fourth type of waste BW9.

Roughly 15% of the hops constituents are retrieved in the beer, and the rest (85%) becomes spent hop material, BW5 [7]. The BW5 waste obtained after the wort boiling process includes majorly insoluble hop materials, condensation products of hop polyphenols, wort proteins, and isomerized hop acids adsorbed on the solid surface [8,9]. The spent hops cannot be used as feed supplements as they contain 2-methyl-3-buten-2-ol, the product of bitter acid degradation with hypnotic–sedative properties. Therefore, spent hops have been used as fertilizers and soil conditioners, as they have high nitrogen content. They are also mixed with spent grain and used as animal fodder [7]. However, several valuable compounds can be recovered from spent hops, such as flavors, saccharides, and organic hop acids. BSY (BW7) is the second major by-product of the brewing industry that has a significant environmental impact as this waste has a large quantity of biomass (1 hl of beer generates 2–4 kg of BSY) [10] and water which makes their management quite difficult for the brewing industry. The BW7 waste is rich in carbohydrates, proteins, amino acids, ash, and vitamins. BSY is also rich in minerals such as calcium, chromium, copper, iron, magnesium, manganese, phosphorus, potassium, selenium, and zinc [7]. Yeast’s mineral content is approximately 5–10% of the cell dry weight [9]. Therefore, these brewery industry wastes are rich sources of several essential compounds that can be recycled efficiently for various applications. However, they are usually mixed with spent grains and supplied as animal fodder or used for laboratory alcohol synthesis.

Metal and metal oxide nanoparticles have been extensively researched and applied due to their unique size-dependent properties. Waste-mediated synthesis of nanoparticles has been explored over the past decade. It has the advantage of decreased use of chemical precursors and waste side-products. Several metal-based nanoparticles such as Ag, Au, Zn, Cu, and Fe have been prepared by this method. Grape stalk waste extract has been used for producing Ag nanoparticles with application in screen-printed electrodes [11]. The reduction of silver ions to obtain silver nanoparticles has been explored using wastes such as orange peel, mango peel, and pineapple extract [12]. Furthermore, tansy fruit extracts have been used to produce silver and gold nanospheres [13]. Additionally, platinum and palladium nanoparticles have been synthesized using lignin, banana peel, tea, and coffee extracts [12]. Nanoparticles of metal oxides such as copper oxide synthesized using brown alga extract have been reported as well [14]. Although a limited number of recent research has reported the synthesis of Ag nanoparticles using beer yeast [15], the unprocessed brewery wastes still have remained unexplored in recycling applications despite being so rich in several valuable compounds.

On the other hand, orthophosphate-based nanoparticles came into the limelight for their photosensitivity in the visible region [16]. The most widely used chemical synthesis method includes a metal source and another source of orthophosphate co-precipitated to form nanoparticles. Template-assisted synthesis is yet another route explored [17]. However, alternative natural-/waste-product-mediated greener synthesis routes for silver orthophosphate nanoparticles have not yet been explored to the best of our knowledge. Moreover, the application of silver phosphates as antimicrobial agents has been limited. As we are on the verge of the ‘post-antibiotic era’ because of significantly increasing antimicrobial resistance, nanoparticles are essential alternatives. They attack the microbes with several different mechanisms, including direct contact with the bacterial cell wall, and therefore, the antimicrobial resistance observed for current antibiotics would not be relevant [18,19]. Therefore, this research focused on the possibility of utilizing waste produced in the brewing industry for the preparation of silver phosphate nanoparticles. This method is a simple, cost-, time-, and energy-efficient way of synthesizing metal phosphate nanoparticles. Herein, nanocomposites of silver were synthesized using two different brewery by-products: filtered wastes from stage 7 (BW7) and stage 5 (BW5). Characterization of the brewery wastes indicated the composition differences between BW7 and BW5. We have further studied the effect of temperature and time of synthesis on nanoparticles’ structure, elemental composition, morphology, and their surface chemical states. The nanoparticles were applied as antibacterial agents against a Gram-negative bacteria strain to understand the influence of their chemical composition on the antibacterial activity.

## 2. Materials and Methods

### 2.1. Materials

Silver nitrate (99.9% purity) was purchased from Avantor Performance Materials S.A. Poland (former POCH S.A., Gliwice, Poland). Brewery waste was provided by Jabłonowo Brewery, Wólka Kossowska, Poland. Brewery wastes and silver nitrate were the precursors for nanoparticle synthesis.

### 2.2. Synthesis of Nanoparticles

The schematic of nanoparticle synthesis is shown in Scheme 1. It was a simple one-pot synthesis procedure. The unprocessed brewery wastes directly from the production line were used for nanoparticle synthesis. The brewery wastes were filtered using 150 mm filter paper. A measure of 50 mL of the filtered brewery waste (BW5 or BW7) was heated over a magnetic stirrer under constant stirring at 100 rpm until the desired temperature (25, 50, and 80 °C) was attained. Subsequently, silver nitrate salt was added to the filtered waste to obtain the concentration of 100 mM. The reaction was carried out at various times (10, 30, or 120 min). The pH of BW7 was 4.85, and that of BW5 was 4.3 at the beginning of the synthesis. The pH of the solutions decreased slightly after synthesis to ~4 (BW7) and ~3.5 (BW5), possibly due to the removal of capping agents from the brewery waste. The solution was then centrifuged at 10,000 rpm in a MPW 351R centrifuge (MPW MED. INSTRUMENTS, Warsaw, Poland) after cooling it for up to 10 min to ~35 °C. The supernatant was discarded, and distilled water was used to wash the nanoparticles two times. Then, the nanoparticles were kept for drying for 24 h at ~40 °C in an air oven. Finally, the powder form of nanoparticles was used for further analyses. The nanoparticles were characterized for their bulk phase and elemental composition and surface morphology and composition. Furthermore, their antimicrobial activity was studied to understand the effect of nanocomposite composition on inhibition and bactericidal efficiency.

### 2.3. Instruments and Analysis Methods

The brewery wastes were examined for the presence of elements, total nitrogen, total polyphenols, total carbohydrates, total fermenting carbohydrates (fructose, glucose, maltose+ sucrose, maltotriose, sulfate (SO_4_^2−^), total organic carbon (TOC), and other chemical compounds. The elemental content of the majority of elements was examined by a conventional combustion method. Cl was examined using the potentiometric–argentometric method by titration with AgNO_3_ standard, whereas the total nitrogen content was determined using the Kjeldahl titration method (PN-A-04018:1975) [20]. The content of total polyphenols was determined using the spectrophotometric method (PN-A-79093-13:2000) [21]. The total carbohydrate content was determined using the spectrophotometric method according to 9.26, 8.14 Analytica EBC, 2.7.3 MEBAK (2013) [22]. The content of total fermenting carbohydrates was determined using high-performance liquid chromatography with refractometric detection (HPLC/RID) and external standards according to 9.27, 8.7 Analytica EBC, 2.7.2 MEBAK (2013) [23]. The sulfate content was determined using high-performance liquid chromatography with conductometric detection (HPLC/CD) using an external standard according to 2.22.1 MEBAK (2013) [24]. The total organic carbon was determined using the spectrometric method according to CLA/SR/26/2012 [25]. The chemical compounds present were determined using high-performance liquid chromatography and mass spectrometry (HPLC-MS).

Fourier-transform infrared (FT-IR) spectra of brewery waste liquid solutions and powdered nanoparticles were recorded under dry nitrogen at atmospheric pressure and under 5 hPa, respectively, using the Fourier Vertex 80 V spectrophotometer equipped with multi-reflection Attenuated Total Reflectance accessory—multi-ATR and DTGS detector (Bruker Inc., Billerica, MA, USA). The spectra were recorded using apparatus parameters such as the full width at half-maximum (FWHM) of 2 cm^−1^ and number of scans 1024. The Empyrean D8 X-ray diffractometer (Bruker Inc., Billerica, MA, USA) was used for recording the powder X-ray diffraction (PXRD) patterns of the polycrystalline nanomaterials. This apparatus was equipped with Bragg–Brentano geometry, an X-ray tube (Cu Kα radiation) with Kβ filters goniometer in a theta–theta vertical system. The identification of chemical compounds and their phases present in the nanoparticles was achieved using X’PertHighscore Plus software. The Nova NanoSEM 450 (FEI, Hillsboro, OR, USA) scanning electron microscope (SEM) recorded images at 10kV high voltage and immersion imaging mode. Energy dispersive X-ray analyzer (EDX) attached to SEM was used for surface elemental mapping. A MiniPal 4 PW4025/00 Energy dispersive X-ray fluorescence (EDXRF) spectrometer (PANanalytical Inc., Malvern, UK) was used to analyze the elemental composition of nanomaterials. The X-ray photoelectron spectroscopy (XPS) measurements were carried out in an ultra-high-vacuum for surface chemical analysis using the ESA-31 electron spectrometer (home-made) [26]. The spectrometer was equipped with a hemispherical electron energy analyzer of a high relative energy resolution of 0.5% without retardation (the retarding ratio (k) can be changed from 2 up to 100), an electron gun (LEG62-VG Microtech), a home-made X-ray excitation source (Al KαX-rays hν = 1486.67 eV), and an Ar^+^ ion source of AG21 (VG Scientific). The XPS spectra were measured in the fixed retarding ratio (FRR) mode (k = 4, 8, 16) at a photon incidence and electron emission angles of 70° and 0°, respectively, with respect to the surface normal of the specimen.

### 2.4. Bacterial Susceptibility Tests

Nanocomposites synthesized after 120 min at 25 and 80 °C were tested for their activity against the Gram-negative strain *Escherichia coli* ATCC 25922. The bacterial strain was grown overnight on Lysogeny broth (LB) agar media (Carl Roth GmbH + Co. KG, Karlsruhe, Germany). An inoculum of the strain was prepared in the Mueller Hinton (MH) broth (Becton, Dickinson and Company, Franklin Lakes, NJ, USA) by adding the colonies from the agar plates to achieve an optical density of 0.1, corresponding to ~10^8^ CFU mL^−1^. The nanoparticles were ultrasonicated using the VCX 130 ultrasonic processor at 30% amplitude for 10 min in the pulse mode to enable uniform dispersion. The pulse mode was used to prevent over-heating of the solution. The nutrient broth and agar media were sterilized in a Varioklav steam sterilizer (HP Medizintechnik GmbH, Oberschleissheim, Germany).

The minimum inhibitory concentration (MIC) of nanoparticles was determined as the primary parameter that would enable further advanced studies. Stock solutions of 1 mg mL^−1^ nanocomposites were prepared in MH nutrient broth containing 10% phosphate buffer saline (PBS). PBS enabled better dispersion of nanocomposites. Serial dilution was performed on a 96-well plate to obtain nanocomposite concentrations between 0.5 and 50 μg mL^−1^. The bacterial strain was then added to the 96-well plates to achieve a final concentration of 5 × 10^5^ CFU mL^−1^. After 24 h, alamarBlue reagent (Life Technologies Europe BV, Bleiswijk, Netherlands) was added. The plates were further incubated for 2 h, after which the absorbance value was measured using the plate reader. After MIC determination, the minimum bactericidal concentration (MBC) was determined by plating the dilutions on agar plates. Concentrations of nanocomposites higher than MIC up to 4 times were contacted with 15 mL bacteria inoculum (10^5^–10^6^ CFU mL^−1^) of *E. coli* for 24 h in an orbital shaker incubator at 220 rpm and 37 °C temperature. A 50 μL solution was taken from the tubes and plated after serial dilutions on agar plates. The lowest nanocomposite concentration that resulted in the absence of colonies on agar plates after 24 h incubation was considered the bactericidal concentration.

Furthermore, time-kill kinetics were observed at sub-MIC, MIC, and MBC concentrations between 0 and 24 h of nanoparticles contact with bacterial inoculum. The time-kill studies were performed by exposing a 5 mL inoculum (10^5^–10^6^ CFU mL^−1^) of *E. coli* to a defined concentration of nanocomposites in 15 mL centrifuge tubes for up to 24 h. The tubes were covered with aluminum foil to prevent the interaction of nanocomposites with light radiation. A 50 μL solution was taken from the tubes and plated after serial dilutions on agar plates. The number of colonies was counted, and the final concentration of *E. coli* was determined in a 1 mL solution. A positive control without nanoparticles was used as a reference for bacteria growth.

## 3. Results

### 3.1. Brewery Wastes Analyses

The composition and elemental analysis of brewery wastes obtained in two production stages: the fifth stage (BW5: wort precipitate) and the seventh stage (BW7: Brewer’s spent yeast) are presented in Table 1. The brewery waste BW7 is richer in nitrogen, polyphenols, and sulfates as compared to BW5. BW5, disparately, has a significant content of carbohydrates and sugars. BW7 is deficient in both carbohydrates and sugars. BW7 also has high potassium, magnesium, sodium, and manganese content, whereas BW5 has significantly more phosphorous content. The influence of these composition changes is on the nanocomposite formation, discussed in detail in the Discussion section. Moreover, small amounts of iron, zinc, and copper can also be seen in brewery wastes. A similar carbon content is detected in both the wastes in total organic carbon (TOC) analysis. As the sucrose content in BW5 and BW7 was negligible, the total fermentable sugars can be equated to reducing sugars present in brewery wastes.

**Figure 1:**
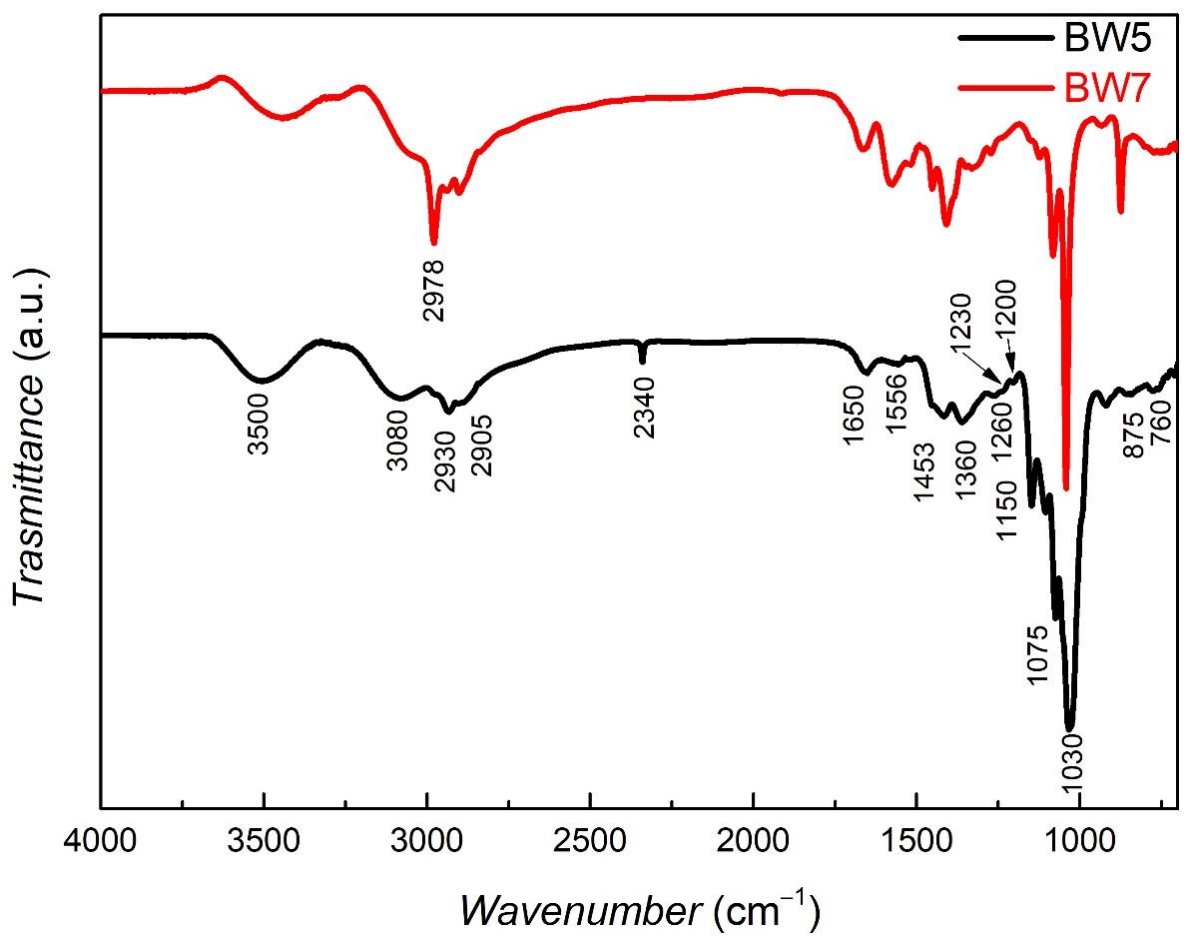
FT-IR spectra of brewery wastes BW7 and BW5.

Significant differences can be observed in the FT-IR spectra of the two brewery wastes, BW7 and BW5 (Figure 1). The broad band between ~3700 and 3300 cm^−1^ is related to strong intramolecular H-bonded O–H stretching vibrations of polyphenols combined with the N–H stretching vibrations for hydrogen-bonded NH groups. The band is more intense and slightly shifted to longer wavelengths for BW5. The broad band at 3080 cm^−1^ can be related to the C–H stretching vibrations for compounds built from carbon atoms of sp^2^ hybridizations. The C–H alkane stretching vibrations can be found in both wastes in the 2990–2850 cm^−1^ region, however, there are additional peaks with a higher intensity in BW7. The peaks at 1670 cm^−1^ and 1550 cm^−1^ can be attributed to amide I (C=O stretching with some N–H bending) and amide II (N–H bending with C–N stretching), respectively [27,28]. The C–H alkane bending vibrations can be observed around 1450 and 1360 cm^−1^. The intense bands at 1260, 1150, 1075, and 1030 cm^−1^ could represent C–O vibrations or –O–H bending of primary, secondary, and tertiary alcohols. These vibrations can also relate to the C–O bending of the C–OH groups in carbohydrates, especially in BW5, as it does not contain fermented alcohols [29]. Below 1000 cm^−1^, the out-of-plane X–H bending (X=C, O, N) or NH_2_ rocking and wagging appear. The sharp peaks usually correspond to non-hydrogen-bonded groups. Therefore, the sharp band at 875 cm^−1^ observed in BW7 could correspond to the alcoholic –O–H bending or NH_2_ rocking and wagging vibrations. The absence of this band in the waste obtained before the fermentation stage, BW5, could be due to the lack of yeast and fermentation products [30]. The significantly lower nitrogen content in BW5 (Table 1) supports this band assignment. The rich spectra for both wastes indicate the possibilities of alcohols, amides, and polyphenols in the composition. The functional groups can also be associated with carbohydrates (region ~1150 to 900 cm^−1^), lipids (region ~2950 to 2850 cm^−1^), and proteins (region ~1690 to 1635 cm^−1^) in the wastes along with polyphenolic compounds [27,28,29,30,31,32,33]. Therefore, these wastes can be used for the synthesis of nanoparticles.

### 3.2. Characterization of Nanocomposites

#### 3.2.1. Crystallography and Phase Analysis

**Figure 2:**
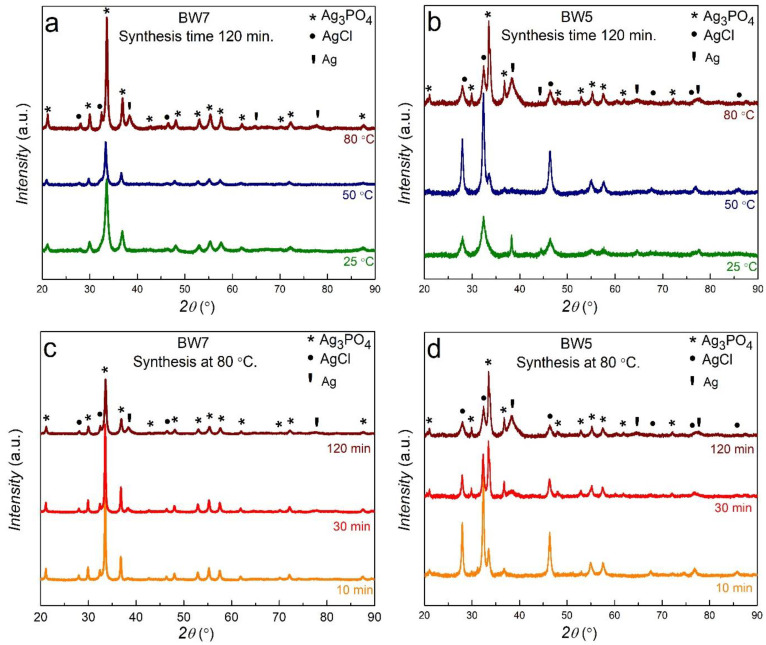
X-ray diffractograms of nanomaterials synthesized at different temperatures and times using brewery wastes (**a**,**c**) BW7 and (**b**,**d**) BW5.

The X-ray diffraction patterns (Figure 2) provided important information on the key differences between the nanoparticles precipitated using different brewery wastes (BW5 and BW7). The PXRD patterns showed reflexes characteristic for Ag_3_PO_4_ (ICDD 98-002-7843), AgCl (ICDD 98-005-6538), and Ag (ICDD 98-060-4629) phases. The phase composition was determined from the most intensive PXRD reflexes representing every phase, i.e., (012) at *2θ* = 33.3° for Ag_3_PO_4_, (002) at 2*θ* = 32.3° for AgCl and (111) at 2*θ* = 38.1° for Ag metal (Ag_met_). The method using the Reference Intensity Ratio (RIR) values with respect to corundum was applied [34]. The Scherrer’s equation using a constant of 0.94 and accounting for the same reflexes of each phase in 2*θ* range of 20°–60° was applied to evaluate the average values of crystallite size and the respective standard deviations, when considering more than one reflex. The results are listed in Table 2.

The brewery waste from stage 7, BW7, yielded silver orthophosphate nanoparticles (Ag_3_PO_4_) (Figure 2a,c, Table 2) with reflections corresponding to the cubic structure with space group P4-3n. The characteristic Ag_3_PO_4_ reflexes are at 2*θ* values 20.9° (011), 29.7° (002), 33.3° (012), 36.6° (112), 47.9° (013), 52.8° (222), 55.1° (023), 57.4° (123), 61.7° (004), 72.0° (124), and 87.4° (234). An additional reflex at 69.98° (024) 2*θ* is found in samples synthesized at 80 °C. The evolution of new reflexes and narrowing of all crystalline reflexes with increasing synthesis temperature indicated that the crystallinity of the phase enhances with increasing temperature. The crystal structure consists of a body-centered cubic lattice formed by regular isolated tetrahedral PO_4_. According to Ma et al. (2016), the Ag atom experiences fourfold coordination by four O atoms [35]. The P atoms have fourfold coordination surrounded by four O atoms, while the O atoms have fourfold coordination surrounded by three Ag atoms and one P atom. The polyhedron structure described by the same group consists of one PO_4_ tetrahedron and three tetrahedral AgO_4_ combined through the corner oxygen. The Ag_3_PO_4_ phase content decreases from 90 to 77.1% when the synthesis temperature is increased up to 80 °C. The crystallite size of these Ag_3_PO_4_ nanoparticles increased with the increasing synthesis temperature from 9.9 ± 2.3 nm to 16.2 ± 3.3 nm.

Minor quantities (10 wt.%) of silver chloride nanoparticles with an average crystallite size of 5.5 ± 3.6 nm (Table 2) have been identified in nanocomposites synthesized at room temperature. The reflexes around 2*θ* values 27.8° (111), 32.3° (002), and 46.3° (022) belong to silver chloride (AgCl). The crystallinity of all three phases, Ag_3_PO_4_, AgCl, and Ag, increases with synthesis temperature. The AgCl nanoparticle phase slightly grows from 10 to 11.8 wt.% and the size of the crystallites grow with increasing temperature. The AgCl content does not significantly increase as BW7 contains a relatively insignificant amount of chlorine (Table 1). The AgCl has a body-centered cubic structure with a space group Fm-3m. When the synthesis temperature increases to 80 °C, Ag_met_ nanoparticles are also formed, indicating that high temperature facilitates the reduction of Ag ions in the salt precursor silver nitrate from +1 to 0 oxidation state. Reflexes of Ag are present at 2*θ* values 38.1° (111), 64.5° (022), and 77.4° (113). The Ag phase steadily increases at 80 °C from 10 to 120 min synthesis time at the expense of the major phase Ag_3_PO_4_. The AgCl and Ag_3_PO_4_ crystallite growth is favored over nucleation with increasing synthesis temperature. The crystallite size of Ag_3_PO_4_ and AgCl increases from 10 to 30 min and then decreases when the synthesis time is increased to 120 min. This unusual trend could indicate that the synthesis time affects two competing mechanisms: nucleation and growth. The size of Ag crystallites decreases with the synthesis time, indicating that nucleation is favored over this phase’s growth.

The stage 5 brewery waste, BW5, diversely, gave rise to the mixed composite of majorly AgCl. The AgCl phase content up to 91.5 wt.% could be obtained at room temperature synthesis. The Ag and Ag_3_PO_4_ nanoparticles were found in minor amounts (Figure 2b,d, Table 2). With time and increasing synthesis temperature, more Ag_3_PO_4_ nanoparticles (up to 30 wt.%) and Ag (up to 28.5 wt.%) were introduced into the composite structure. The crystallographic structures of the various nanoparticles in these composites were similar to those observed for nanoparticles obtained using BW7. The crystallite size of Ag_3_PO_4_ nanoparticles increases, and that of Ag nanoparticles decreases with increasing temperature. Temperature favors the growth of Ag_3_PO_4_ crystallites and nucleation of Ag crystallites. Ag nanoparticle growth at the expense of AgCl could be the reason for the smaller crystallite size of AgCl at 80 °C. The Ag and Ag_3_PO_4_ phases grow at the expense of AgCl with time at 80 °C synthesis temperature. The crystallites’ size of these two phases also increases with time; however, that of AgCl decreases. This effect is similar to that observed with synthesis temperature for AgCl crystallites. Synthesis time favors the growth of nanocrystallites of minor phases such as Ag and Ag_3_PO_4_. Silver oxides could not be observed in PXRD diffractograms, probably because their content is below the detection limit.

#### 3.2.2. Elemental and Morphological Analysis

The elemental content analysis of the samples was performed by energy dispersive X-ray fluorescence (EDXRF). The nanomaterials obtained by using brewery wastes BW5 and BW7 contained silver (Ag), chlorine (Cl), phosphorus (P), and sulfur (S). Table 3 shows the calculated weight percentage of these elements in the samples. The area under the peak for Ag was the highest, confirming that Ag metal-based nanoparticles are formed. The Ag content is almost similar in all the BW7 nanomaterials. The slight differences are related to the growth of minor amounts of Ag phases (up to 11.1 wt.%) with temperature and time (Table 2). The content of P decreases with increasing time and temperature, which is also coherent with Ag metal nanoparticle formation at higher temperatures observed in PXRD. The content of Cl and S is almost constant in all the BW7 nanomaterials. Only a minor amount of Cl is observed (~1%) coherent with the low AgCl phase content (up to 12 wt.%) (Table 2).

In BW5 nanomaterials, the content of Ag increases from 86.5 to 92.2 wt.% with increasing synthesis temperature. The growth of a larger amount of Ag_met_ nanoparticles in the composite (up to 28.5 wt.%) observed in PXRD is concurrent with this increase. Additionally, at 80 °C synthesis temperature, the yield is increased with time as the Ag content increases from 87.6% to 92.2%. The amount of Cl in BW5 nanocomposites decreases with increasing temperature and time of synthesis as the formation of Ag metal nanoparticles is favored. This observation is coherent with the decrease in the AgCl phase observed in PXRD (Table 2). The S present as traces in all the samples is included in the organic layer stabilizing the nanoparticles proved further by XPS analysis.

**Figure 3:**
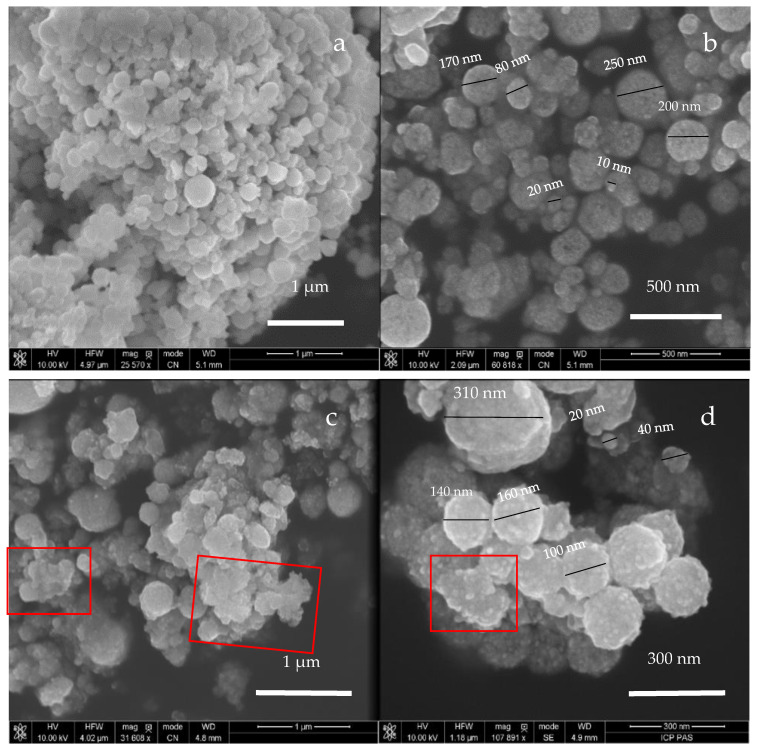

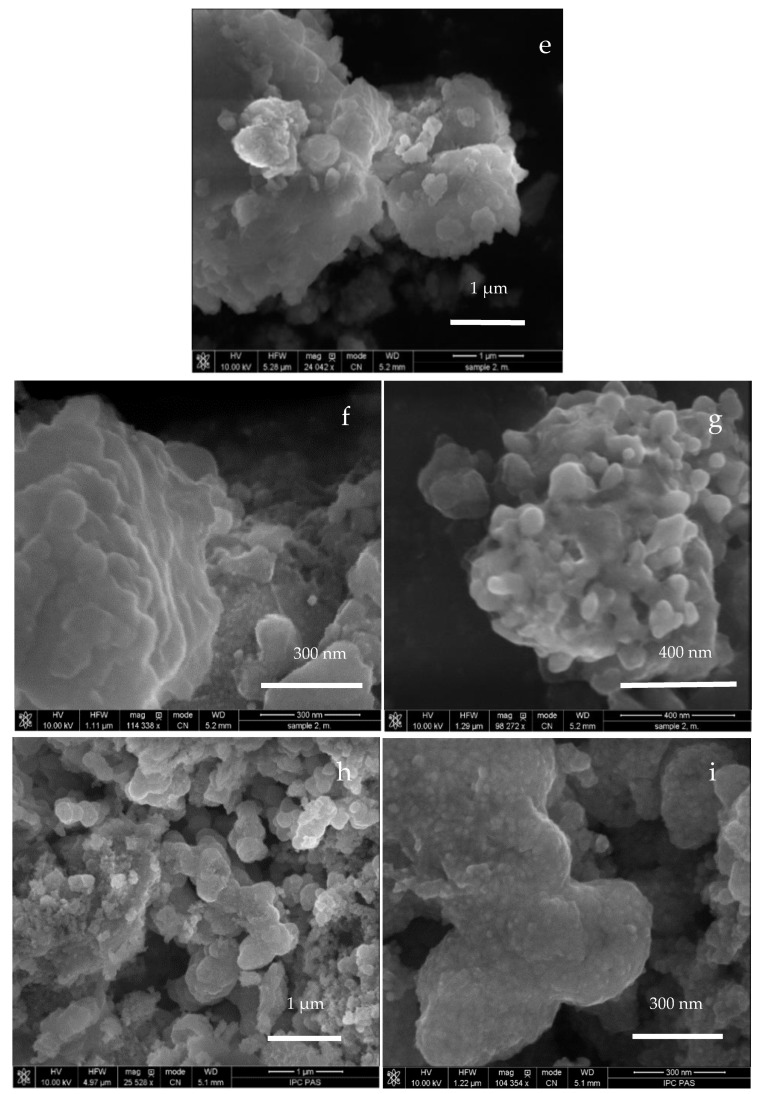
SEM images of nanomaterials synthesized after 120 min using brewery wastes BW7 at (**a**,**b**) 25 °C (BW7Ag1), (**c**,**d**) 80 °C (BW7Ag3) and BW5 at (**e**–**g**) 25 °C (BW5Ag1), (**h**,**i**) 80 °C (BW5Ag3).

The morphology of Ag_3_PO_4_-rich sample BW7Ag1 obtained using BW7 at 25 °C (Figure 3a,b) shows globular structures which appear to be made of several smaller balls. As AgCl and Ag increase in the structures with increasing synthesis temperature (BW7Ag3), the flakey aggregated layers are fused with balls as observed in the areas marked in red (Figure 3c,d). The size of these globular structures ranges from ~10 to 350 nm in BW7 nanocomposites. BW5 nanocomposite at 25 °C (BW5Ag1) shows aggregated layers, characteristic of AgCl along with some small balls and globules (Figure 3e–g). With increasing Ag_3_PO_4_ in the structure, fused morphology similar to BW7 nanocomposites is observed (Figure 3h,i). In Figure 3i, the particles observed have a surface similar to the Ag_3_PO_4_ balls in BW7 nanocomposites. The elemental mapping in Figure S1a–d (Supplementary Information-SI) indicates that the elemental distribution of Ag, Cl, and P on the surface of the nanocomposites is sufficiently homogeneous. This indicates that the nanoparticles’ morphological changes in shapes and sizes are due to the incorporation of certain phases in the structure; however, these phases are not present independently but rather as a mixed phase composite.

#### 3.2.3. Chemical Analysis

**Figure 4:**
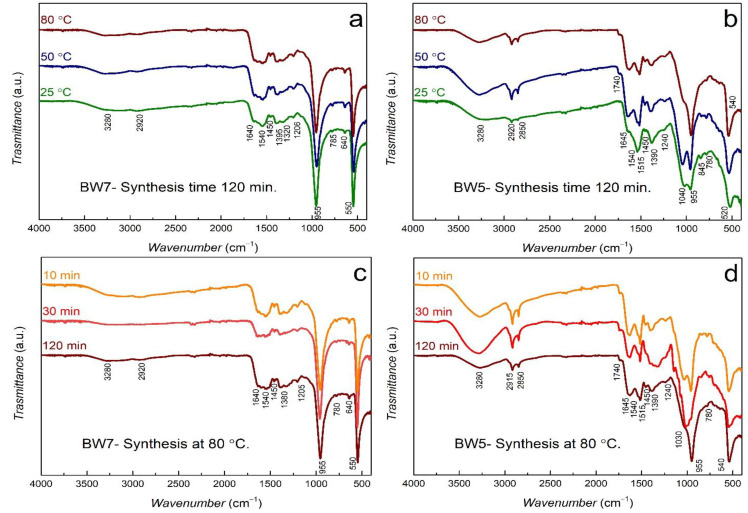
FT-IR spectra of nanomaterials synthesized at different temperatures and times using brewery wastes (**a**,**c**) BW7 and (**b**,**d**) BW5.

Both sets of nanocomposites show a broad band between 3500 and 3000 cm^−1^, representing the stretching vibrations of the hydrogen-bonded OH group of polyphenols, although the relative intensities of these peaks vary depending on the nanocomposite (Figure 4). This broad band can also be attributed to the N–H stretching vibration of amide. This vibration is stronger in BW5 nanocomposites, indicating a higher amount of surface-bound hydroxyl groups and other hydrogen-bonded functional groups. The shift in this band to lower frequencies in nanoparticles compared to its position in brewery wastes (Figure 1) is related to the complexation of the functional groups to nanoparticles. In addition, several sharp peaks observed between 2840 and 3000 cm^−1^ belong to symmetric and asymmetric C–H stretching vibrations from alkanes in various chemical environments. These stretching vibrations are also significantly intense in BW5 nanocomposites, but not so in the case of BW7 nanocomposites. Interestingly, the C-H-containing compounds are better visible in the BW7 waste than in BW5 (Figure 1). Moreover, the dominant band in this region for BW7 waste is 2978 cm^−1^ indicating the presence of compounds with –CH_3_ moieties. On the other hand, BW7 nanocomposites exhibit only very weak bands in this region. BW5 nanocomposites show important bands in this region, and the dominant peaks at 2915–2920 and 2850 cm^−1^ are indicative of mainly –CH_2_– moieties presence. The amide I and II vibrations observed at 1640 cm^−1^ and 1540 cm^−1^ related to peptide-protein binding are also from the brewery wastes. These vibrations are intense in nanocomposites synthesized using BW5 and very weak in BW7 nanocomposites. This contradicts the situation observed in the case of wastes, where the intensity of those bands is weaker in the case of BW5 than BW7 (Figure 1). Considering the nanoparticle composition, one could infer that the protein-peptide adsorption is more predominant in the case of AgCl nanoparticles than in the case of Ag_3_PO_4_ ones. The intense band between 1030 and 1040 cm^−1^ representing C–O vibration is observed in the BW5 nanocomposites representing the functional groups in BW5 (Figure 1). The capping of BW5 nanocomposites shows a higher number of functional groups in the FT-IR analysis, indicating that these materials’ surface composition is richer than the BW7 nanocomposites. The functional group vibrations observed for these nanoparticles are from the brewery wastes BW7 and BW5, indicating a successful capping of the nanoparticles. Furthermore, the shifts in the frequencies of these vibrations in nanoparticles (Figure 4) compared to that in the brewery wastes (Figure 1) indicate a high level of complexation of the functional groups with the nanoparticles. Not all functional groups aid in the capping of nanoparticles, and depending on the brewery waste, different groups have been attached to the nanoparticles’ surface. It seems that the binding of protein–peptide, as well as polyphenols, is more preferred on the surface of AgCl nanoparticles.

Additional vibrations relating to Ag_3_PO_4_and silver oxides can also be observed in the nanocomposites. The peak observed for BW7 and BW5 nanocomposites at frequencies of ~520–550 cm^−1^ represent Ag‒O stretching modes. The band around 550 cm^−1^ is also attributed to a bending vibration of O=P‒O, whereas the band at ~960 cm^−1^ to the asymmetric stretching of the OP–O bridge [35,36,37]. These modes of PO_4_^3−^ are visibly sharp for BW7 nanocomposites at all temperatures and time, providing evidence of Ag_3_PO_4_ as the major phase. Characteristic vibration modes for Ag_3_PO_4_ become more prominent with the increasing temperature and time for BW5 nanocomposites, indicating the growth of Ag_3_PO_4_ in the nanocomposite. There is also a shift in the vibration frequency from 520 to 540 cm^−1^ as phosphate groups grow in the BW5 nanocomposites with increasing synthesis temperature. The lower frequency of vibration for BW5 nanocomposite at 80 °C, as compared to BW7 nanocomposite, is because Ag_3_PO_4_ is a minor phase in it. Therefore, it has a more complex environment around it that influences its vibration.

The survey XPS spectra of BW7 and BW5 nanomaterials are shown in Figure S2. The quantification of the surface atomic content in the specimens (Table 4) was carried out accounting for the area under Ag 3d, C 1s, O 1s, N 1s, P 2p, S 2p, and Cl 2p photoelectron peaks originating from the XPS experiments after using Tougaard’s correction for the background from inelastically scattered electrons [38]. The XPS MultiQuant software [39,40] evaluated surface atomic content, accounting for Scofield photoionization cross-sections [41], electron inelastic scattering, and analyzer transmission function.

The binding energy (BE) values calibration was performed for all the photoelectron spectra, i.e., Ag 3d_5/2–3/2_, C 1s, O 1s, N 1s, S 2p, P 2p, and Cl 2p, considering the Ag 3d_5/2–3/2_ predominant component resulting from the PXRD patterns (Table 2).

The surface of BW7 and BW5 nanocomposites shows the presence of Ag (Ag 3d_5/2–3/2_), C (C 1s), O (O 1s), N (N 1s), Cl (Cl 2p), P (P 2p), and S (S 2p). The elemental content depends on nanomaterial synthesis conditions and the brewery waste. The ratio of C to Ag is larger for BW5 nanocomposites, indicating a thicker carbon overlayer on silver nanoparticles composite (Figure S3a). At higher temperatures, the larger ratio of C to O for BW5 nanocomposites indicates oxygen deficiency of its carbon overlayer, contrary to BW7 nanocomposites (Figure S3b). Simultaneously, with increasing temperature, the ratio of Ag to O increases. This increase is relatively lower for BW5 nanocomposites, indicating the formation of a higher amount of silver oxides and the incorporation of a higher amount of carbon-oxygen groups when BW5 is used (Figure S3c).

The BE values for all elements present were evaluated after binding energy scale calibration and averaging the respective values for BW7 and BW5 nanocomposites prepared at different temperatures and synthesis times. These BE values confirm the presence of the chemical compounds. Nitrogen (N 1s BE = 399.4 ± 0.1 eV) indicates an amino group (C–NH_2_) [42] present in protein structures. Chlorine (Cl 2p BE = 198.6 ± 0.1 eV) is identified as AgCl [42]. In BW7 nanocomposites with Ag_3_PO_4_ as the predominant phase, the phosphorous peak (P 2p BE = 132.9 ± 0.1 eV) indicates phosphate (PO_4_), phosphite (PO_2_), or phosphine (P=N=P) groups [42]. In BW5 nanocomposites with AgCl as the predominant phase, phosphorous peak (P 2p BE = 133.3 ± 0.1 eV) indicates hydrogen phosphate (HPO_4_), diphosphate (P_2_O_7_), triphosphate (P_3_O_10_), dioxide diphosphorus (H_4_P_2_O_2_), or phosphate in the organic matrix, e.g., methyl phosphate (PO_3_CH_3_) [42]. The chemical state of sulfur indicates two forms, which depend on the nanoparticle synthesis temperature and time. The chemical form of sulfur at BE = 162.9 ± 0.1 eV indicates sulfur linked in an organic environment, whereas the chemical form at BE = 168.0 ± 0.1 eV indicates sulfonyl (SO_2_), sulfonate (SO_3_), or sulfate (SO_4_) groups [42]. This last form of sulfur starts occurring between 50 and 80 °C.

Comparison of elementary weight content in bulk and at the surface resulting from EDXRF and XPS analyses for Ag, P, Cl, and S, where the XPS results from Table 4 are normalized to 100 percent, is provided in Figure S4. No significant differences in elemental bulk and surface content are observed. The increasing Ag content is observed with increasing temperature and synthesis time, contrary to decreasing P, Cl, and S content.

The Ag 3d_5/2–3/2_, C 1s, and O 1s spectra were fitted to Gaussian–Lorentzian asymmetric components after Tougaard inelastic background subtraction [38] using the XPSPeakfit software [43]. The following binding energy (BE) values for Ag 3d_5/2_ spectrum were considered in the fitting procedure of Ag 3d_5/2–3/2_ spectra to determine the surface content of Ag chemical states: 367.8 eV for Ag_3_PO_4_ [44,45]; 368.1 eV for AgCl [46]; 368.3 eV for Ag_met_ [47,48]; and 367.3 eV for Ag oxidized structures such as Ag_2_O, AgO, AgOH [47,48]. The fitting proceeded with constant ratios of full width at half maximum (FWHM) for different Ag phases. The presence of Ag_2_O was reported at BE shifted towards smaller values with respect to Ag_met_ by 0.5–0.6 eV (BE = 367.7–367.8 eV) and AgO and AgOH structures at BE shifted respectively by 0.9–1.0 eV (BE = 367.4–367.3 eV) [47,48]. Additionally, Ag-glucose structures were reported at BE shifted towards smaller BE with respect to Ag_met_ by about 1 eV [49]. To obtain the accurate fitting results, the additional peak at BE smaller than that for Ag oxidized structures were included, where its BE value resulted from the fitting procedure. The fitting results of Ag 3d_5/2–3/2_, C 1s, and O 1s are presented in Figure S5a–c, respectively.

The results from the fitting of the weight content of Ag chemical states are listed in Table 5. No Ag_met_ is observed at the surface of BW7 and BW5 nanocomposites. The surface shows Ag_3_O_4_, AgCl, and Ag oxidized states (Ag_2_O, AgO, AgOH) and the peak at BE = 364.09 ± 0.11 eV (BW7) and BE = 363.8 ± 0.3 eV (BW5), which is interpreted as AgO-polymer state. This AgO-polymer chemical state resulting from AgO-carbohydrate/polyphenol/amino structures linked through hydrogen bonds is manifested in BE shifted towards smaller BE values than Ag oxides and AgOH. Its BE value depends on carbohydrates, polyphenols, and total nitrogen content forming amino groups in brewery wastes. The higher content of carbohydrates and sugars in BW5 nanocomposites (Table 1), where the synthesis conditions using the BW5 waste promote AgCl formation, leads to a larger BE shift with respect to Ag_met_ and provides a smaller ratio of AgO-polymer to Ag oxidized forms (Table 5). The respective BE shift is smaller in BW7 nanocomposites, where the synthesis conditions predominantly led to the Ag_3_PO_4_ chemical state. The larger ratio of AgO-polymer to Ag oxidized forms indicates the more facile formation of AgO-polymer state over Ag oxides and AgOH. This suggests different organometallic structures linking Ag oxides and organic material originating from amino groups, polyphenols, carbohydrates, and sugars present in both wastes and differentiated by their content (Table 1).

The comparison of Ag phase weight content resulting from the PXRD and XPS analyses as temperature and time of synthesis dependence for BW7 and BW5 nanocomposites is shown in Figure S6a–d respectively. The XPS results show the content normalized to 100 percent. The BW7 nanocomposites containing the predominant Ag_3_PO_4_ phase exhibited decreased Ag_3_PO_4_ phase content accompanied by increasing AgCl phase. The Ag oxides, AgOH, and AgO-polymer phases also decreased with increasing synthesis temperature (Figure S6a). The synthesis at 80 °C decreased Ag_3_PO_4_ phase content, accompanied by increasing AgCl, Ag oxides/AgOH, and stable AgO-polymer forms on the surface with synthesis time (Figure S6c). The BW5 nanocomposites containing primarily AgCl phase exhibited a decrease of AgCl, accompanied by increasing Ag_3_PO_4_, Ag oxides/AgOH, and AgO-polymer forms (Figure S6b,d). In BW7 nanocomposites, the AgO-polymer content is remarkably higher than Ag oxides/AgOH, contrary to BW5 nanocomposites. The bulk and surface content of AgCl and Ag_3_PO_4_ phases are in reasonable agreement; however, the surface shows a large amount of Ag oxidized structures such as Ag oxides, AgOH, and Ag oxides linked to polyphenols, carbohydrates and/or amino groups via hydrogen bonds forming a kind of organometallic structure.

For the C 1s spectra, the following BE resulting from the applied calibration using Ag 3d_5/2–3/2_ spectra were applied: 284.4 ± 0.1 eV for C sp^2^ hybridizations, 285.3 ± 0.1 eV for C sp^3^ hybridizations, 286.5 ± 0.1 eV for hydroxyl group C–OH, 287.5 ± 0.1 eV for carbonyl group–C=O [46,50]. The carboxyl group COOH is not present in the investigated samples. For the O 1s line, the following BE were obtained: 531.2 ± 0.2 eV for C=O, 532.5 ± 0.1 eV for C–O and 529.9 ± 0.2 for Ag oxidized states [50,51]. The atomic contents of oxygen groups (C–OH, C=O) and Ag– oxidized chemical state resulting from the fitting of O 1s spectra are in agreement with their respective atomic contents resulting from the fitting of C 1s (C–OH, C=O) and Ag 3d_5/3–3/2_ (Ag–O) spectra. The carbon overlayer thickness on Ag nanoparticles was evaluated from Ag 3d_5/2–3/2_ spectra using Tougaard Quases-Analyze software, where the surface morphology parameters are reflected in the shape of the inelastic background in the vicinity of photoelectron transition and its intensity [52]. Assuming a Buried Layer (BL) model of uniform overlayer and inelastic mean free path (IMFP) of Ag _3d5/2–3/2_ photoelectron attenuated in carbon overlayer, i.e., 3.1 nm [53], the subtraction of the inelastic background provides the value of overlayer thickness (Figure S7a,b). The results of C 1s spectra fitting representing the content of different carbon forms and carbon overlayer thickness in nanocomposites are presented in Table 6. The overlayer thickness dependence with temperature and synthesis time for BW7 and BW5 nanocomposites agree with the C to Ag ratio (Figure S3a), indicating a relatively thinner overlayer for BW7 nanocomposites. Simultaneously, the carbon overlayer of BW7 nanocomposites is oxygen-enriched (Figure S3b) due to the larger content of Ag-polymer chemical state and contains smaller content of Ag oxides (Figure S3c) than BW5 nanocomposites.

The BW7 and BW5 nanocomposites contain different hydroxyl and carbonyl groups content due to differences in synthesis conditions (temperature and time) (Table 6). The capping of BW5 nanocomposites shows a larger number of functional carbon-oxygen groups, indicating that these materials’ surface composition is richer than the BW7 nanocomposites. This observation is in agreement with the FT-IR analysis (Figure 4). The surface of BW7 nanocomposites shows C–OH groups predominantly, whereas BW5 nanocomposites contain C–OH and C=O groups in all samples. In both nanomaterials, 50 °C synthesis temperature favors increasing C sp^2^ at the expense of C sp^3^. The synthesis time (10–120 min.) at 80 °C leads to carbon amorphization not affecting the content of C–OH and C=O groups remarkably. The synthesis temperature (25–80 °C) repairs C sp^3^ defects and builds C–OH groups. Amorphization of carbon in nanoparticles is related to increasing thickness of overlayers, whereas carbonization is related to decreasing thickness. The overlayer thickness in BW5 nanocomposites exceeds that in BW7 nanocomposites, which may also be due to a larger content of carbohydrates and fermentable sugars (Table 1) taking part in the formation of this overlayer. For BW5 nanomaterials, the thickness increases with increasing synthesis time from 10 to 30 min and then decreases with further synthesis time, whereas the synthesis temperature does not change it remarkably. No remarkable change is observed in overlayer thickness with the time of synthesis for BW7 nanocomposites.

#### 3.2.4. Bacterial Susceptibility Testing

Susceptibility of a Gram-negative strain *Escherichia coli* (*E. coli* ATCC 25922) to nanocomposites synthesized after 120 min reaction (BW7Ag1, BW7Ag3, BW5Ag1, and BW5Ag3) were studied. These nanocomposites were chosen to include a broad spectrum of composition. The minimum inhibitory concentration (MIC) of nanocomposites synthesized using BW7 at 25 and 80 °C was 9.375 μg mL^−1^ (Table 7). However, the nanoparticles synthesized using BW5 at 25 and 80 °C showed varied influence on the inhibition of bacterial strain. The nanocomposite synthesized at 25 °C (BW5Ag1) was slightly more lethal for the bacterial strain than the nanocomposite synthesized at 80 °C (BW5Ag3).

**Figure 5:**
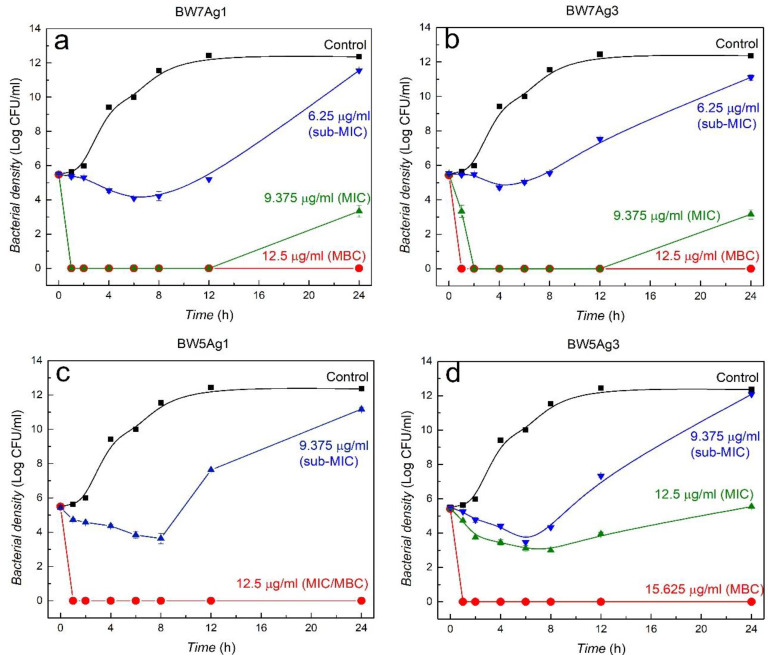
Time-kill curves of E. coli exposed to four different nanocomposites at different concentrations. Nanocomposites synthesized after 120 min reaction time using brewery wastes BW7 at (**a**) 25 °C (BW7Ag1) and (**b**) 80 °C (BW7Ag3) and BW5 at (**c**) 25 °C (BW5Ag1) and (**d**) 80 °C (BW5Ag3).

BW7 nanocomposites (BW7Ag1 and BW7Ag3) at MIC concentration (9.375 μg mL^−1^) inhibited bacterial growth entirely up to 12 h, and then slight growth was observed between 12 and 24 h (Figure 5a,b). It could indicate a tolerance developed in the bacterial strain to the nanoparticles or the inability of the nanoparticles to continue the inhibition mechanism. BW5 nanocomposite at 25 °C (BW5Ag1) completely killed the tested bacteria at MIC (Figure 5c). Therefore, at 12.5 μg mL^−1^, no colonies were observed on agar plates even after 24 h of contact between nanoparticles and the bacterial inoculum. However, at concentrations lower than 12.5 μg mL^−1^, the nanocomposite inhibition capacity is unable to outpace the bacteria growth beyond 8 h. Contrastingly, the BW5 nanocomposite at 80 °C (BW5Ag3) only marginally inhibited the bacterial growth at MIC concentration (12.5 μg mL^−1^) (Figure 5d). The BW5Ag3 nanocomposite killed the inoculum only at a much higher concentration of 15.635 μg mL^−1^, indicating that this nanocomposite synthesized at a higher temperature using BW5 has the least antimicrobial activity. The sub-MIC concentration time-kill curves for all nanocomposites indicate that they are able to inhibit bacterial growth up to 8 h; however, they are outpaced by the bacterial cell reproduction speed.

**Figure 6:**
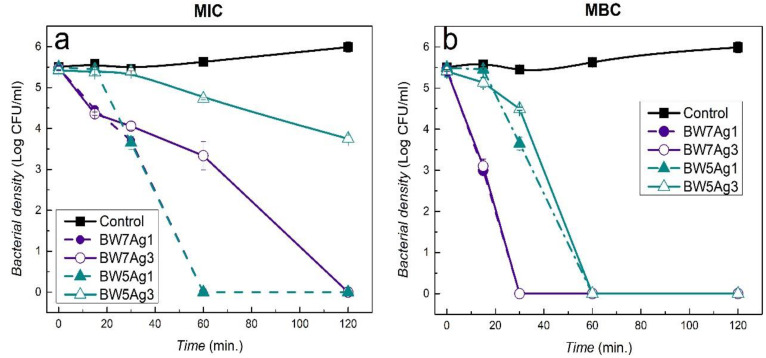
Time-kill kinetics of E. coli exposed to (**a**) MIC and (**b**) MBC concentrations of nanocomposites synthesized after 120 min reaction time using brewery wastes BW7 and BW5 at 25 (BW7Ag1 and BW5Ag1) and 80 °C (BW7Ag3 and BW5Ag3).

Time-kill kinetics indicated the rate at which the nanocomposites affect the bacterial strain *E. coli* (Figure 6). Low-temperature synthesis has led to more potent nanocomposites for antibacterial activity. The nanocomposites synthesized using BW7 and BW5 at 25 °C (BW7Ag1 and BW5Ag1) take up to 1 h to kill the inoculum at MIC concentrations (Figure 6a). The BW7 nanocomposite at 80 °C (BW7Ag3) takes 2 h for killing, and the BW5 nanocomposite at 80 °C (BW5Ag3) does not exhibit complete killing even after 2 h contact time. This indicates that BW7Ag1 affects the bacteria at the fastest rate. At minimum bactericidal concentrations (MBC), all nanoparticles effectively kill the inoculum in 1 h. However, BW7 nanocomposites (BW7Ag1 and BW7Ag3) affect at a faster rate as compared to BW 5 nanocomposites (BW5Ag1 and BW5Ag3) (Figure 6b).

## 4. Discussion

The temperature and time of synthesis affected the relative compositions of the three phases of silver, namely Ag_3_PO_4_, AgCl, and Ag_met_, in the nanocomposites. The lack of chlorine in the BW7 and the abundance of phosphorus-based compounds cause silver phosphate formation. The presence of yeast and a nitrogen-rich environment that is highly deficient in sugars can also be instrumental in the growth of silver phosphate. Contrastingly, BW5 is rich in phosphorous and chlorine; therefore, competing reactions favored the growth of majorly AgCl, and Ag_3_PO_4_ is incorporated as the minor component. The spontaneous formation of AgCl results from the exchange reaction between silver nitrate and a source of halide present in the brewery wastes. Therefore, in the presence of high amounts of Cl in the BW5 waste, AgCl is obtained in major quantities. After the formation of AgCl, the excess silver nitrate follows another exchange reaction with the phosphate source leading to Ag_3_PO_4_. As the Cl content in BW7 is very low, the major phase in BW7 nanocomposites is predominantly Ag_3_PO_4_ [54]. The conversion of silver salt by both brewery wastes led to incorporating higher amounts of the minor contents in the nanocomposite structure with increasing synthesis temperature and time. Increasing the synthesis temperature can promote reactions that may not be spontaneous under room temperatures, leading to several parallel reactions yielding a composite structure. The growth of Ag_met_ in BW7 nanocomposites is favored only when the temperature is increased to 80 °C (BW7Ag3). At lower temperatures, the formation of Ag_3_PO_4_ is predominant. Reduction with BW5 also yields Ag_met_ nanoparticles with smaller crystallites size as a function of increasing synthesis temperature. The most favored reaction at 25 °C is the formation of AgCl. Although Ag_3_PO_4_ and Ag_met_ are also obtained at lower synthesis temperatures (BW5Ag1), the growth of the phases is promoted at 80 °C (BW5Ag3). With increasing synthesis temperature, higher aggregation of particles is also observed for BW7 and BW5 nanocomposites. The reducing agents present in BW7 and BW5 are activated at higher temperatures, resulting in the reduction of Ag ions from +1 to 0 oxidation state.

Amino acids, carbohydrates, polyphenols, and sugars are well-known reducing agents responsible for reducing silver ions to silver metal and capping of nanoparticles [55,56]. However, different compounds present in BW7 and BW5 are responsible for nanoparticle synthesis. Sugars are known to act as potent reducing agents under alkaline conditions [57]. The added effect of hydroxyl radicals in silver reduction is also evident at alkaline pH. Therefore, the reducing capacity of these agents is not prominent in our synthesis as the brewery wastes are acidic. The reduction by polyphenols is also affected by the system’s pH, which impacts the nanoparticle size and shape. The reduction by polyphenol at acidic pH leads to larger size nanoparticles [58]. Therefore, the acidic pH in our system led to the formation of a small amount of large-sized Ag_met_ nanoparticles in the composite structure primarily due to polyphenol-induced reduction.

Amino acids and proteins contain carbonyl groups that exhibit a strong affinity towards metal nanoparticles [59]. Therefore, they are some of the critical capping agents. However, although proteins are present in both wastes, the overlayer of BW5 nanocomposites contains protein groups, but the BW7 nanocomposites overlayer shows only relatively small intensity vibrations, which can be attributed to proteins. This indicates that the surface of Ag_3_PO_4_ and AgCl plays a major role in determining the organic groups that will form a part of the overlayer. The protein–peptides preferentially attach to nanocomposites with major AgCl in the structure. Several organic functional groups have participated in the capping. Therefore, the organic overlayer is rich in different C chemical states (Csp^2^, Csp^3^, C–O, C=O) and contains B, N, and S. The overlayer of BW5 nanocomposites is enriched with C=O functional group. BW5 nanocomposites also have a thicker overlayer capping than BW7 nanocomposites. The overlayer has the largest thickness when the synthesis temperature is 50 °C. The high temperature appears to be facilitating the attachment of organic groups to the nanoparticle surface. However, this overlayer in BW5 nanocomposites formed at higher temperatures is less oxygen-enriched than in BW7 nanocomposites.

Although the mechanism of interaction of Ag nanoparticles on cells is complicated and till now not fully understood, there are few factors that simultaneously affect the cell: (i) adhesion on the surface of bacterial wall and membrane leading to membrane disruption, (ii) penetration into the cell, (iii) damage of intracellular organelles and biomolecules, (iv) oxidative stress due to formation of reactive oxygen species, and (v) modulation of signal transduction pathways [60,61]. The adhesion mechanism may result from the presence of negatively charged lipopolysaccharides forming different bonds between Ag^+^ ions and electrostatic interaction. Penetration may proceed via water-filled channels called porins or those formed due to membrane disruption proving its better permeability. The binding of Ag^+^ ions with the phosphorous and sulfur groups in the DNA results in condensation of DNA, preventing further replication. This ultimately prevents cell reproduction leading to microorganism death [60]. The results obtained with the AgCl and Ag_3_PO_4_ nanoparticles would pave the way to a more detailed study the dominant factors in the antimicrobial activity of silver and its compounds.

Using both the brewery wastes, the nanocomposites synthesized at 25 and 80 °C after 120 min are effective antimicrobial agents. However, nanocomposites synthesized using BW7 (BW7Ag1 and BW7Ag3) are more effective at inhibiting the Gram-negative *E. coli* strain growth. These Ag nanoparticles are characterized by the smallest nanoparticle size, which may facilitate their penetration via cell membrane inside a cell before cell disruption due to adhesion, resulting in faster damage to cell organelles and biomolecules. Moreover, BW7Ag1 affects the bacteria faster than any other nanocomposite. It is noteworthy that the concentration of Ag^+^ ions in solution from Ag_3_PO_4_ (~1.3 × 10^−4^ M) is an order of magnitude higher as compared to that from AgCl (~1.3 × 10^−5^ M) when calculated using the solubility products (*K*_sp_) of Ag_3_PO_4_ (8.89 × 10^−17^) and AgCl (1.77 × 10^−10^) at 25 °C [62].

Therefore, the relatively larger Ag_3_PO_4_ content and consequently higher content of Ag^+^ ions in the structure could be responsible for the higher activity of BW7 nanocomposites. Additionally, the surface of BW5 nanocomposites contains different forms of phosphorous other than phosphate in contrast to BW7 nanocomposites. Moreover, the activity of BW7Ag1 could be higher also because of the smaller size, resulting in a higher surface area for adhesion. Therefore, the larger availability of Ag^+^ ions and higher surface area could be responsible for the better antimicrobial activity of nanocomposites synthesized using BW7. Alternatively, the surface of silver phosphate nanoparticles could be more toxic for the bacterial cell than AgCl and Ag_met_. Moreover, a thinner overlayer capping (<20 Å) with a higher amount of hydrolyzed forms (CO^−^ and OH^−^) of C–OH groups could facilitate a better contact of the nanoparticles with the bacterial cells. The incorporation of Ag_met_ and increase of the crystallites size of Ag_3_PO_4_ and AgCl in nanocomposite synthesized at 80 °C (BW7Ag3) has decreased its inhibition rate compared to nanocomposite synthesized at 25 °C. Furthermore, BW5 nanocomposites (BW5Ag1 and BW5Ag3) exhibit a relatively lower antimicrobial activity, and both have higher amounts of Ag_met_ in the structure. This indicates that Ag_met_ is not the most effective component for the inhibition of bacterial growth. The large size of Ag_met_ particles could be the reason for this lowering of the antibacterial effect as they would not be able to penetrate the cell wall. Additionally, the Ag_met_ increases at the expense of Ag_3_PO_4_ or AgCl, thereby decreasing the active material content. Therefore, the nanocomposite synthesized at 80 °C (BW5Ag3) has the least antimicrobial activity as it has the highest Ag_met_ content in its structure. Additionally, these nanocomposites have a thicker overlayer capping (>20 Å) and are enriched with sp^2^ hybridized carbon groups that could be crucial in decreasing the interactions between the bacterial cells and nanoparticles. Overall, nanocomposite systems could be better than nanoparticles of a single-phase because of their combined synergistic effect against bacterial strains, thereby decreasing the probability of bacterial resistance.

Successful eco-friendly one-pot syntheses of Ag nanocomposites containing silver phosphate in a short time and low temperature were performed. Brewery wastes were used as agents to obtain the nanocomposites. It has to be noted that the nanocomposite characteristics are reproducible with repeated synthesis. The obtained nanocomposites can be applied as antimicrobial agents in air and water disinfection by filtration and disinfecting contaminated surfaces. Further studies are being carried out to evaluate the biocompatibility of these nanocomposites, enabling the application of these materials in the food and medicine industries.

## 5. Conclusions

- Nanocomposites of mainly Ag_3_PO_4_ and minor amounts of AgCl and Ag_met_ were obtained using BW7. BW5, disparately, converted the silver salt to mainly AgCl with minor amounts of Ag_3_PO_4_ and Ag_met_. The reduction of Ag^+^ ions is promoted only at higher temperatures.
- Nanocomposites with different ratios of Ag_3_PO_4_, AgCl, and Ag_met_ can be obtained by varying the synthesis temperature and time. The highest Ag_3_PO_4_ content (90 wt.%) was obtained using BW7 in room temperature synthesis for 120 min.
- The organic surface capping was rich in functional groups observed in the brewery wastes. The thickness of the organic layer was ~15–40 Å. BW7 nanocomposites contain a thinner capping than BW5 nanocomposites.
- Increasing the synthesis temperature led to the incorporation of higher amounts of the minor components in the nanocomposite structures.
- The nanocomposites differ in their inhibition and bactericidal activity depending on their composition and surface organic overlayer. BW7 nanocomposites exhibited a better antibacterial activity.

## 6. Patents

The Institute of Physical Chemistry of the Polish Academy of Sciences has applied for a Polish provisional patent (P.433717/PK7364KZ).

## Figures and Tables

**Table 1:** Composition analysis of brewery wastes BW7 and BW5.

Analysis	Subtype	Units	Brewery Wastes	
			BW7	BW5
Total nitrogen		mg L^−1^	7454.5	975.8
Total polyphenols			181.2	92.65
Total sulfates			193.03	119.65
Total carbohydrates			4500	156700
Fermentable sugars	Total		1600	115,800
	Fructose		700	2100
	Glucose		300	12,500
	Maltose + sucrose		500	76,500
	Maltotriose		100	24,700
Elemental content	K	mg L^−1^	2710 ± 460	657 ± 111
	P		97 ± 3.9	525 ± 20
	Cl		<4	365 ± 1.0
	Mg		228.3 ± 38.8	125.9 ± 21.4
	Ca		55.0 ± 8.3	69.3 ± 10.4
	Na		64.0 ± 6.4	58.0 ± 5.8
	Mn		1.64 ± 0.13	0.73 ± 0.06
	Fe		0.8 ± 0.11	0.3 ± 0.04
	Zn		2.6 ± 0.36	0.43 ± 0.06
	Cu		0.32 ± 0.03	0.08 ± 0.01
	Al		0.01 ± 0.01	0.01 ± 0.01
	Ni		<0.05	<0.05
Total organic carbon		%	7.24	7.78

**Table 2:** Phase content and nanocrystallites size of BW7 and BW5 nanomaterials.

	Synthesis temp., °C	Synthesis Time, min.	PXRD Analysis					
			Ag Phase Content (wt.%)			Nanocrystallites Size (nm)		
			Ag_3_PO_4_	AgCl	Ag_met_	Ag_3_PO_4_	AgCl	Ag_met_
BW7Ag1	25	120	90.0	10.0	-	9.9 ± 2.3	5.5 ± 3.6	-
BW7Ag2	50		88.7	11.3	-	15.5 ± 4.2	7.5	-
BW7Ag3	80		77.1	11.8	11.1	16.2 ± 3.3	16.5 ± 5.9	7.3
BW7Ag4	80	30	79.2	12.0	8.8	25.3 ± 4.0	26.2 ± 5.2	12.2
BW7Ag5	80	10	86.6	10.8	2.6	22.7 ± 3.8	21.0 ± 8.3	15.1
BW5Ag1	25	120	5.9	91.5	2.6	4.8	4.0 ± 0.5	18.1
BW5Ag2	50		13.8	85.7	0.5	5.6	8.1 ± 0.8	14.0
BW5Ag3	80		30.0	41.5	28.5	12.1 ± 6.2	6.7 ± 0.4	3.2
BW5Ag4	80	30	28.5	46.0	25.6	15.9 ± 6.2	10.2 ± 1.7	1.4
BW5Ag5	80	10	13.1	86.9	-	11.8 ± 0.7	11.9 ± 1.0	-

**Table 3:** Elemental composition of BW7 and BW5 nanomaterials.

Sample	Synthesis temp., °C	Synthesis Time, min.	XRF Analysis			
			Elemental Composition (wt.%)			
			Ag	P	Cl	S
BW7Ag1	25	120	93.5	4.8	1.0	0.8
BW7Ag2	50		93.7	4.3	1.1	0.8
BW7Ag3	80		94.4	3.9	1.0	0.8
BW7Ag4	80	30	94.4	4.0	1.0	0.6
BW7Ag5	80	10	93.8	4.2	1.2	0.8
BW5Ag1	25	120	86.5	5.3	7.7	0.5
BW5Ag2	50		88.4	2.9	8.1	0.6
BW5Ag3	80		92.2	3.0	4.4	0.5
BW5Ag4	80	30	91.6	2.9	5.0	0.6
BW5Ag5	80	10	87.6	2.9	8.8	0.6

**Table 4:** Surface weight composition of BW7 and BW5 nanocomposites by XPS.

Sample	Synthesis temp., °C	Synthesis Time, min.	XPS Analysis						
			Surface Elemental Composition (wt.%)						
			C	O	Ag	N	P	S	Cl
BW7Ag1	25	120	24.0	14.0	50.7	5.0	3.0	1.3	2.0
BW7Ag2	50		24.6	14.7	50.5	4.4	2.5	1.4	1.9
BW7Ag3	80		19.5	12.5	59.3	3.3	3.4	1.4	0.6
BW7Ag4	80	30	19.6	11.6	61.5	3.3	2.5	1.1	0.4
BW7Ag5	80	10	20.6	12.7	57.4	3.9	3.6	1.2	0.6
BW5Ag1	25	120	25.7	15.6	46.8	2.6	4.1	1.3	3.9
BW5Ag2	50		33.3	12.9	43.2	2.8	3.6	1.0	3.2
BW5Ag3	80		26.9	11.2	48.8	2.3	3.5	4.2	3.1
BW5Ag4	80	30	60.2	19.1	18.0	1.2	0.9	-	0.6
BW5Ag5	80	10	41.2	14.2	36.2	2.6	2.3	0.7	2.8

**Table 5:** Comparison of surface Ag chemical state content in BW7 and BW5 nanocomposites.

Sample	Synthesis temp., °C	Synthesis Time, min.	Ag Chemical State (wt.%)—Ag 3d_5/2–3/2_			
			AgO-polymer	Ag_2_O, AgO, AgOH	Ag_3_PO_4_	AgCl
			BE (eV) 364.09 ± 0.11 (BW7) 363.8 ± 0.3 (BW5)	BE (eV) 367.30	BE (eV) 367.8	BE (eV) 368.1
BW7Ag1	25	120	3.35	0.25	42.75	4.35
BW7Ag2	50		4.21	-	40.72	5.57
BW7Ag3	80		5.81	0.49	45.52	7.48
BW7Ag4	80	30	5.98	0.7	49.35	5.47
BW7Ag5	80	10	5.57	-	47.51	4.32
BW5Ag1	25	120	3.82	2.32	2.74	37.92
BW5Ag2	50		3.52	2.51	0.71	36.47
BW5Ag3	80		3.72	7.01	14.73	23.34
BW5Ag4	80	30	0.88	0.57	3.0	13.56
BW5Ag5	80	10	3.09	-	4.36	28.76

**Table 6:** Comparison of surface C chemical state content and overlayer thickness in BW7 and BW5 nanocomposites.

Sample	Synthesis temp., °C	Synthesis Time, min.	C Chemical State (wt.%)—C 1s				XPS QUASES-Tougaard BL Model Overlayer Thickness (Å)
			C sp^2^	C sp^3^	C–OH	C=O	
			BE (eV) 284.4 ± 0.1	BE (eV) 285.3 ± 0.1	BE (eV) 286.5 ± 0.1	BE (eV) 287.5 ± 0.1	
BW7Ag1	25	120	9.78	8.96	0.55	4.71	20.7
BW7Ag2	50		14.2	3.92	3.76	2.72	24.2
BW7Ag3	80		11.37	4.64	3.49	-	18.0
BW7Ag4	80	30	12.46	4.11	3.03	-	17.5
BW7Ag5	80	10	13.0	3.88	3.52	-	17.5
BW5Ag1	25	120	10.36	9.57	2.03	3.74	23.0
BW5Ag2	50		24.38	2.87	2.87	3.18	24.5
BW5Ag3	80		17.60	4.04	2.13	3.13	23.9
BW5Ag4	80	30	41.75	7.52	7.52	3.41	37.0
BW5Ag5	80	10	31.12	3.93	2.86	3.29	32.5

**Table 7:** Minimum inhibitory concentration (MIC) of nanocomposites synthesized using BW7 and BW5.

Sample	Synthesis temp., °C	Synthesis Time, min.	MIC (μg mL^−1^)
			*Escherichia coli*
BW7Ag1	25	120	9.375
BW7Ag3	80		9.375
BW5Ag1	25		12.5
BW5Ag3	80		15.625

## Data Availability

The data are available in the data storage systems in the Research group 18 at the Institute of Physical Chemistry Polish Academy of Sciences, Warsaw, Poland.

## Supplementary Materials

The following are available online at https://www.mdpi.com/article/10.3390/nano11102659/s1, Figure S1a: Elemental mapping of BW7Ag1 nanocomposite. Figure S1b: Elemental mapping of BW7Ag3 nanocomposite. Figure S1c: Elemental mapping of BW5Ag1 nanocomposite. Figure S1d: Elemental mapping of BW5Ag3 nanocomposite. Figure S2: The XPS survey spectra of BW7 and BW5 nanomaterials synthesized at different temperature and time conditions. Figure S3a: Comparison of C to Ag weight ratio for BW7 and BW5 nanomaterials dependent on synthesis (a) temperature and (b) time at 80 °C. Figure S3b: Comparison of C to O weight ratio for BW7 and BW5 nanomaterials dependent on synthesis (a) temperature and (b) time at 80 °C. Figure S3c: Comparison of Ag to O weight ratio for BW7 and BW5 nanomaterials dependent on synthesis (a) temperature and (b) time at 80 °C. Figure S4: Comparison of elementary weight composition resulting from EDXRF and XPS spectra of nanomaterials synthesized at different temperatures and times using brewery wastes (a,c) BW7 and (b,d) BW5. Figure S5a: The XPS Ag 3d5/2–3/2 spectra recorded from BW7 and BW5 nanomaterials synthesized at different temperature and time conditions fitted using Gaussian–Lorentzian asymmetric functions to different atomic chemical states. Figure S5b: The XPS C 1s spectra recorded from BW7 and BW5 nanomaterials synthesized at different temperature and time conditions fitted using Gaussian–Lorentzian asymmetric functions to different atomic chemical states. Figure S5c: The XPS O 1s spectra recorded from BW7 and BW5 nanomaterials synthesized at different temperature and time conditions fitted using Gaussian–Lorentzian asymmetric functions to different atomic chemical states. Figure S6: Comparison of weight and normalized phase content resulting from XRD and XPS spectra, respectively, in nanomaterials synthesized at different temperatures and time using brewery wastes (a,c) BW7 and (b,d) BW5. Figure S7a: Results of Ag 3d5/2/3-2 spectra analysis using QUASES-Analyze software and Buried Layer (BL) model for BW7 nanomaterials at different synthesis temperatures and times at 80 °C. Figure S7b: Results of Ag 3d5_/2/3-2_ spectra analysis using QUASES-Analyze software and Buried Layer (BL) model for BW5 nanomaterials at different synthesis temperatures and times at 80 °C.
