# Supplementary material for: Valorization of Brewery Wastes for the Synthesis of Silver Nanocomposites Containing Orthophosphate
Source: Nanomaterials (Basel). 2021 Oct 10;11(10):2659. doi: 10.3390/nano11102659 (PMC8539218; doi:10.3390/nano11102659)
Supplement: Supplementary file 1 [file nanomaterials-11-02659-s001.zip › Suplementary Material-MDPI revised vf.pdf]

Supplementary Information

Valorization of Brewery Wastes for the Synthesis of Silver Nanocomposites Containing Orthophosphate

Alcina Johnson Sudagar [1,*,†], Neha Venkatesh Rangam [1,*,†], Artur Ruszczak [1], Paweł Borowicz [1], József Tóth [2], László Kövér [2], Dorota Michałowska [3], Marek Ł. Roszko [3], Krzysztof R. Noworyta [1] and Beata Lesiak [1]

[1] Institute of Physical Chemistry, Polish Academy of Sciences, Kasprzaka 44/52, 01-224 Warsaw, Poland; aruszczak@ichf.edu.pl (A.R.); pborowicz@ichf.edu.pl (P.B.); knoworyta@ichf.edu.pl (K.R.N.); blesiak-orlowska@ichf.edu.pl (B.L.)
[2] Institute for Nuclear Research, BemTér 18/c, H-4026 Debrecen, Hungary; toth.jozsef@atomki.hu (J.T.); kover.laszlo@atomki.hu (L.K.)
[3] Institute of Agriculture and Food Biotechnology—State Research Institute, ul. Rakowiecka 36, 02-532 Warsaw, Poland; dorota.michalowska@ibprs.pl (D.M.); marek.roszko@ibprs.pl (M.Ł.R.)
* Correspondence: sudagaralcinajohnson@gmail.com or asudagar@ichf.edu.pl (A.J.S.); nehavr25@gmail.com (N.V.R.)
† These authors contributed equally to this work.

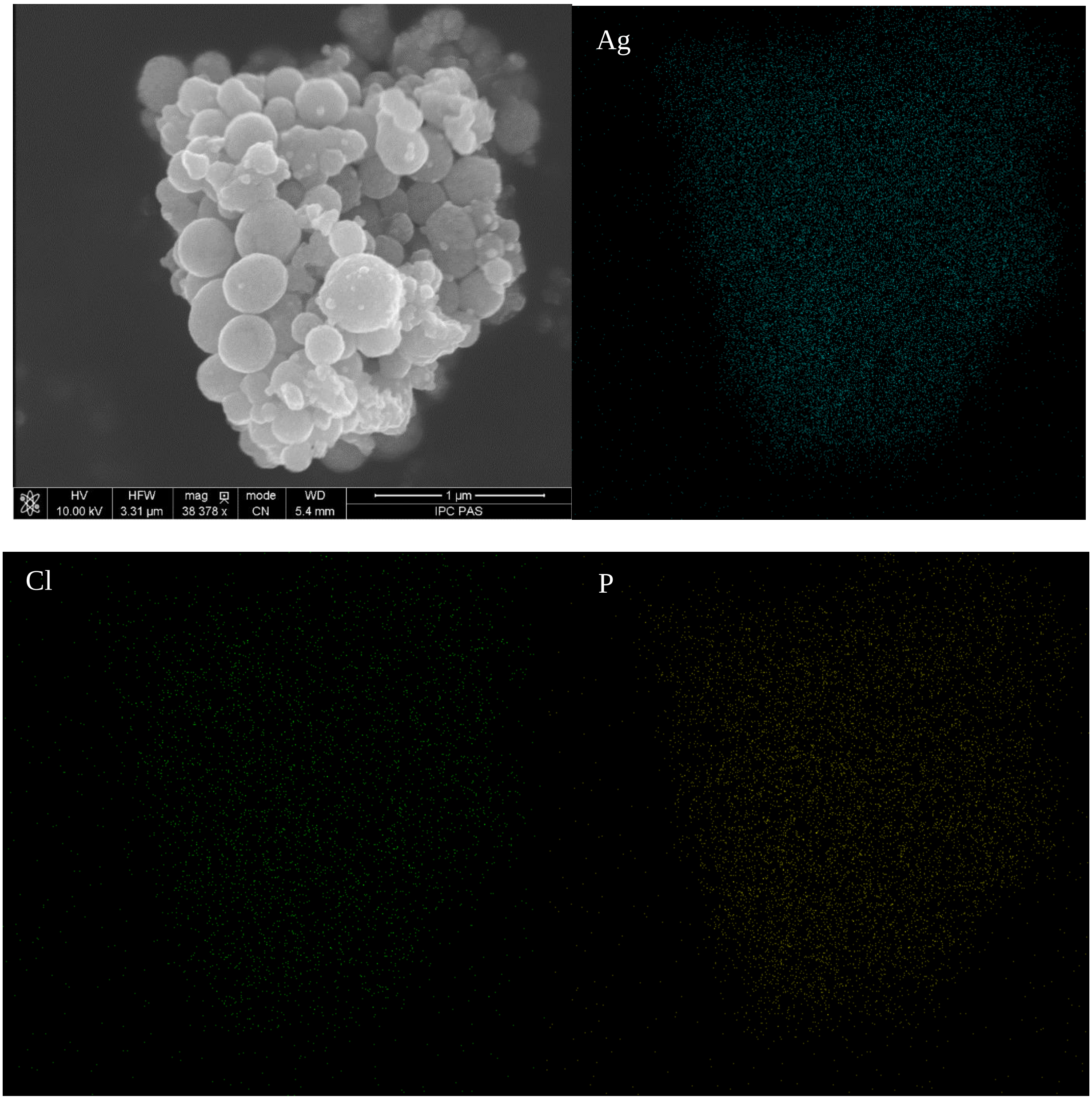

Figure S1a. Elemental mapping of BW7Ag1 nanocomposite.

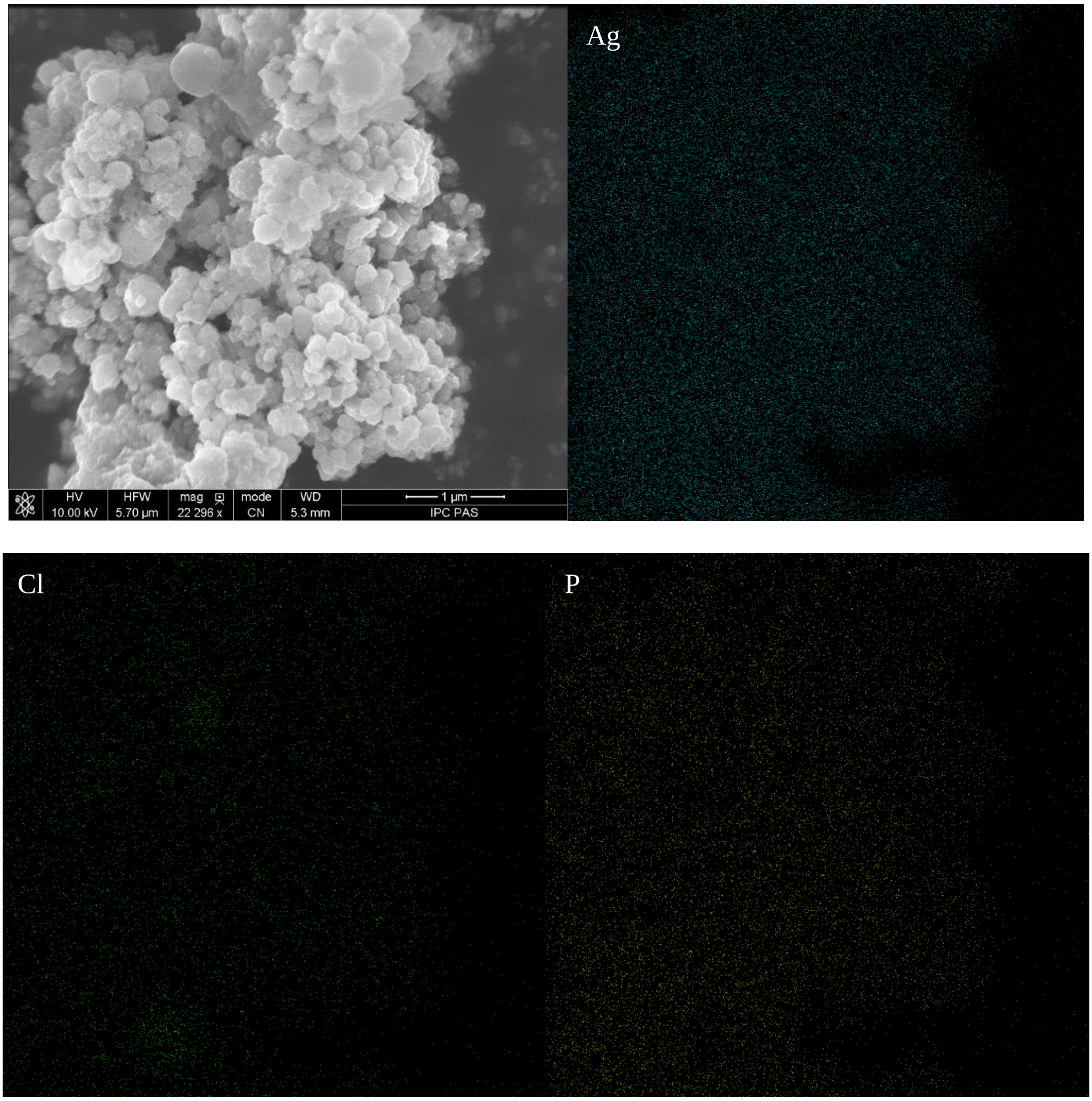

Figure S1b. Elemental mapping of BW7Ag3 nanocomposite.

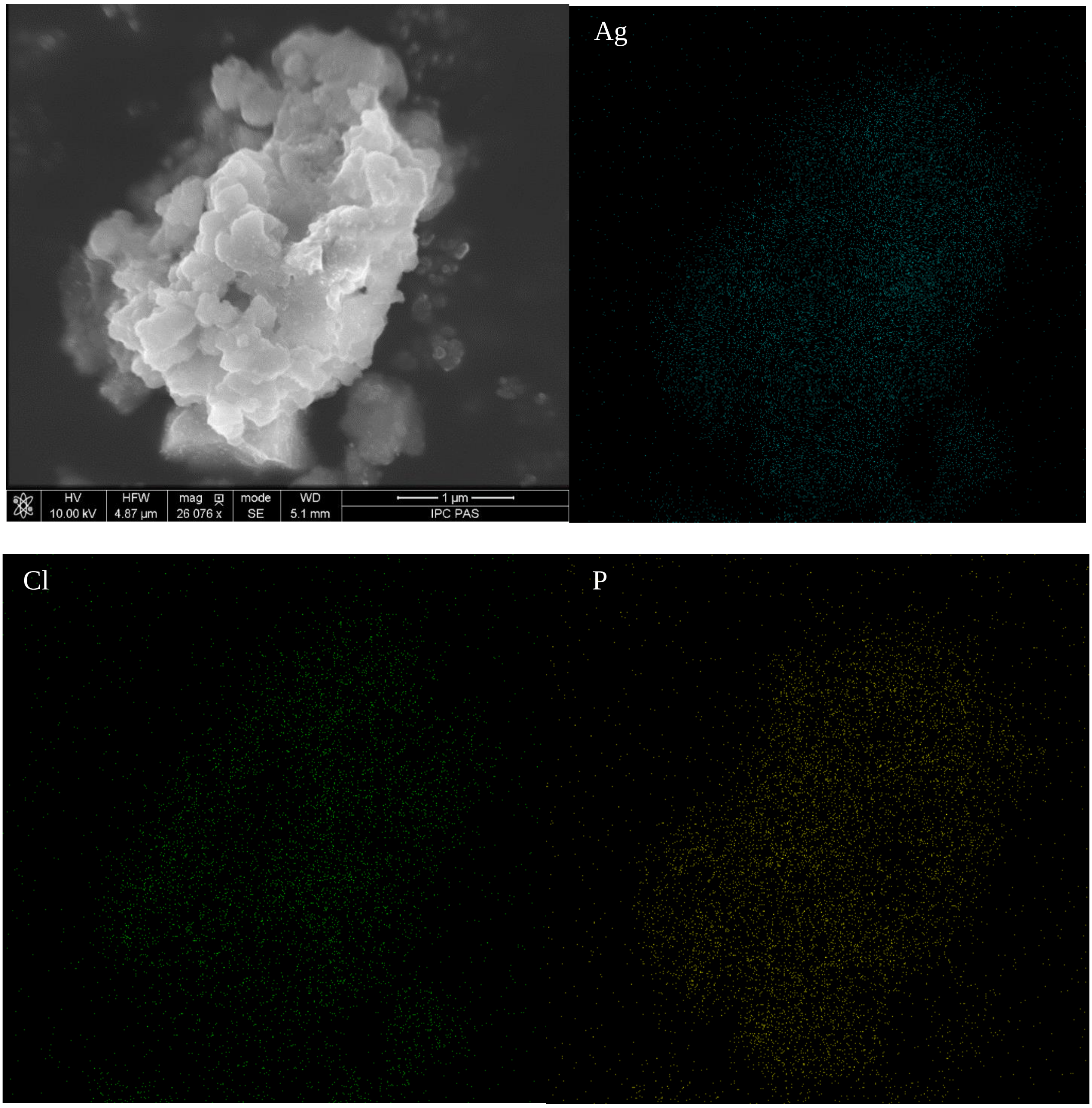

Figure S1c. Elemental mapping of BW5Ag1 nanocomposite.

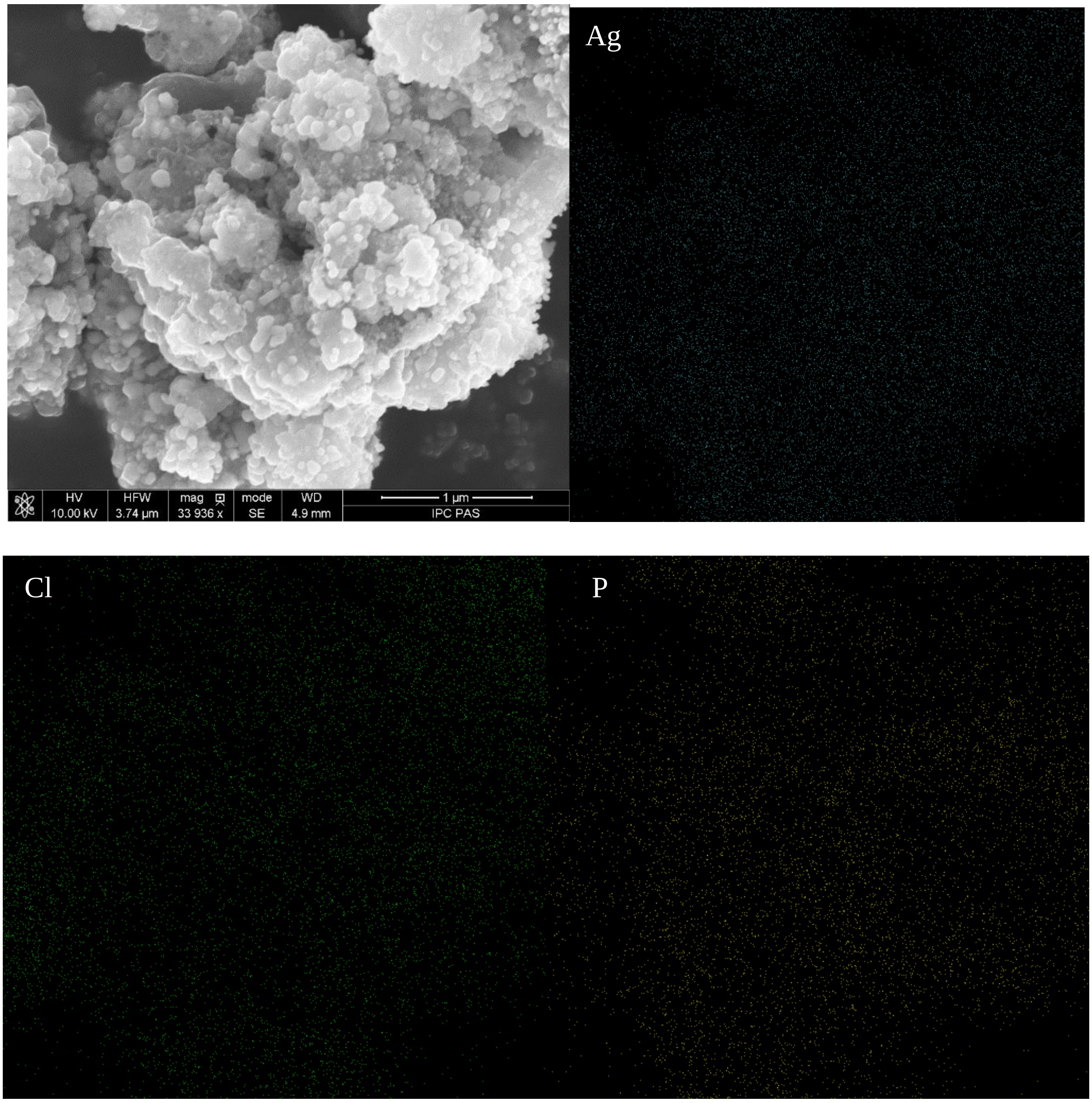

Figure S1d. Elemental mapping of BW5Ag3 nanocomposite.

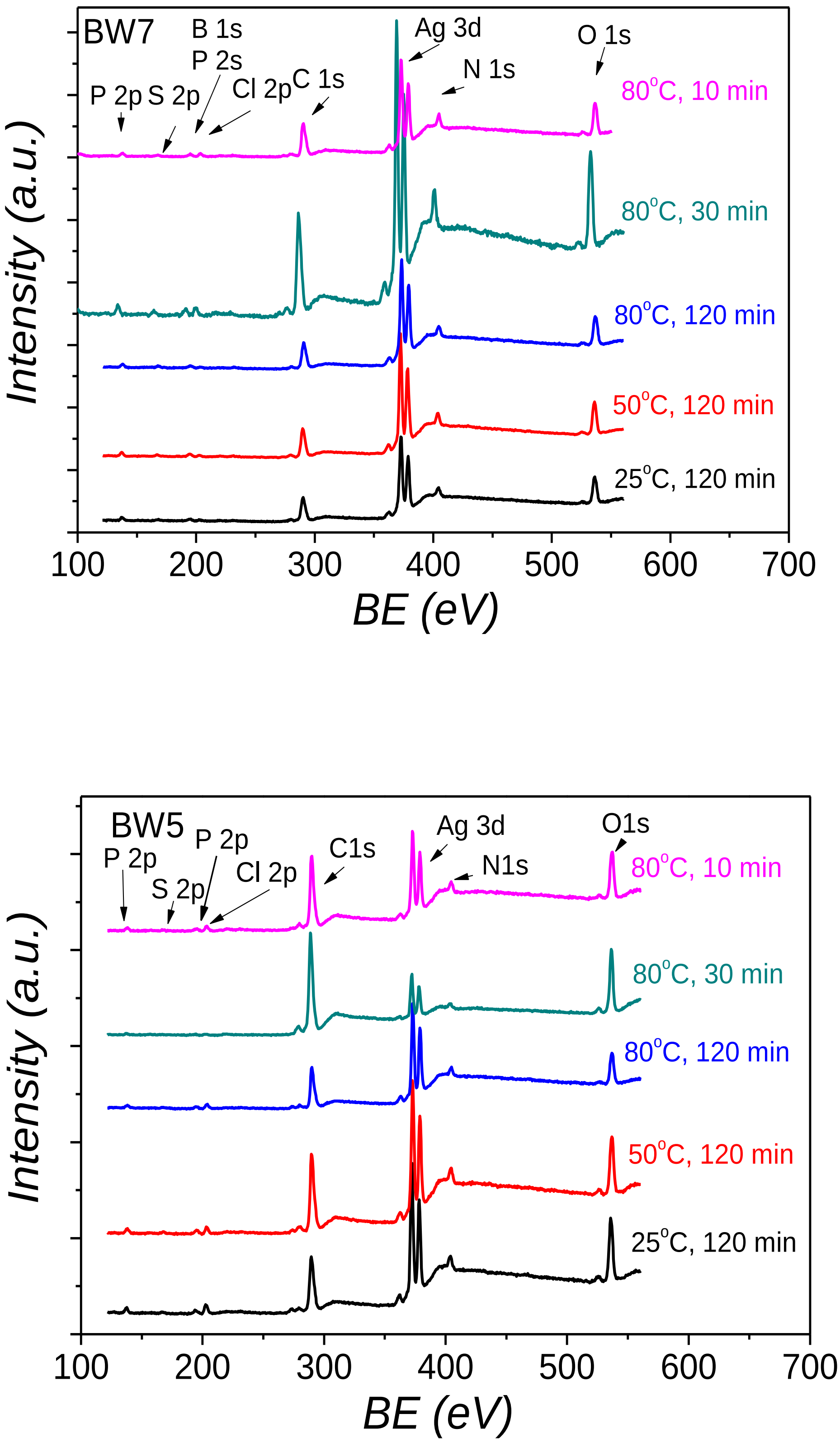

Figure S2. The XPS survey spectra of BW7 and BW5 nanomaterials synthesized at different temperature and time conditions.

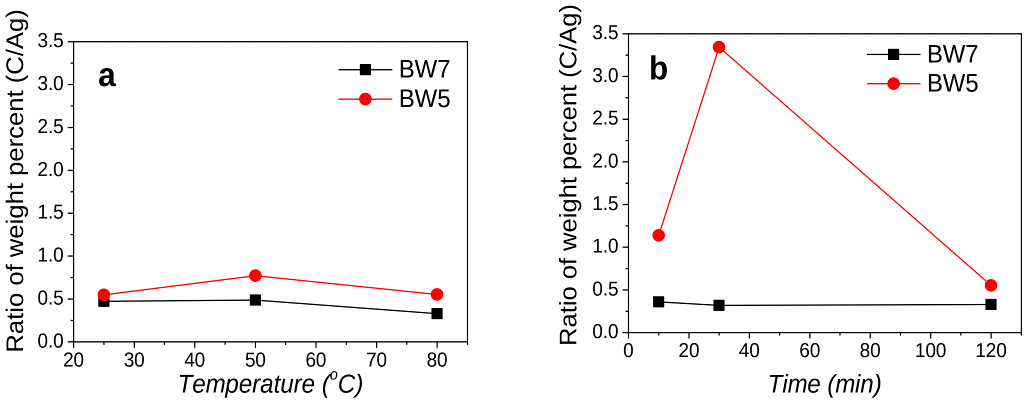

Figure S3a. Comparison of C to Ag weight ratio for BW7 and BW5 nanomaterials dependent on synthesis (a) temperature and (b) time at 80 °C.

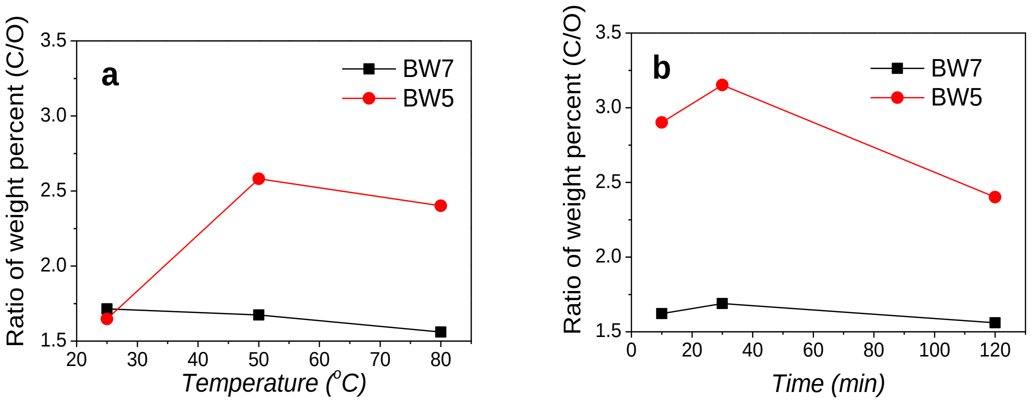

Figure S3b. Comparison of C to O weight ratio for BW7 and BW5 nanomaterials dependent on synthesis (a) temperature and (b) time at 80 °C.

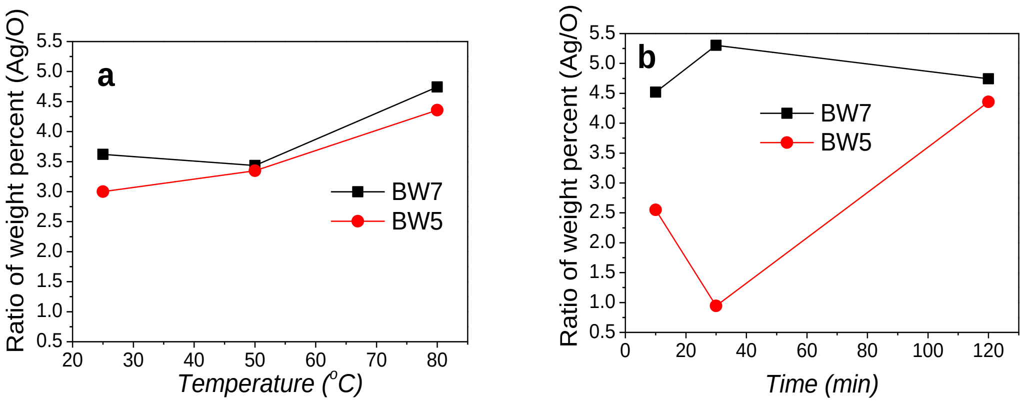

Figure S3c. Comparison of Ag to O weight ratio for BW7 and BW5 nanomaterials dependent on synthesis (a) temperature and (b) time at 80 °C.

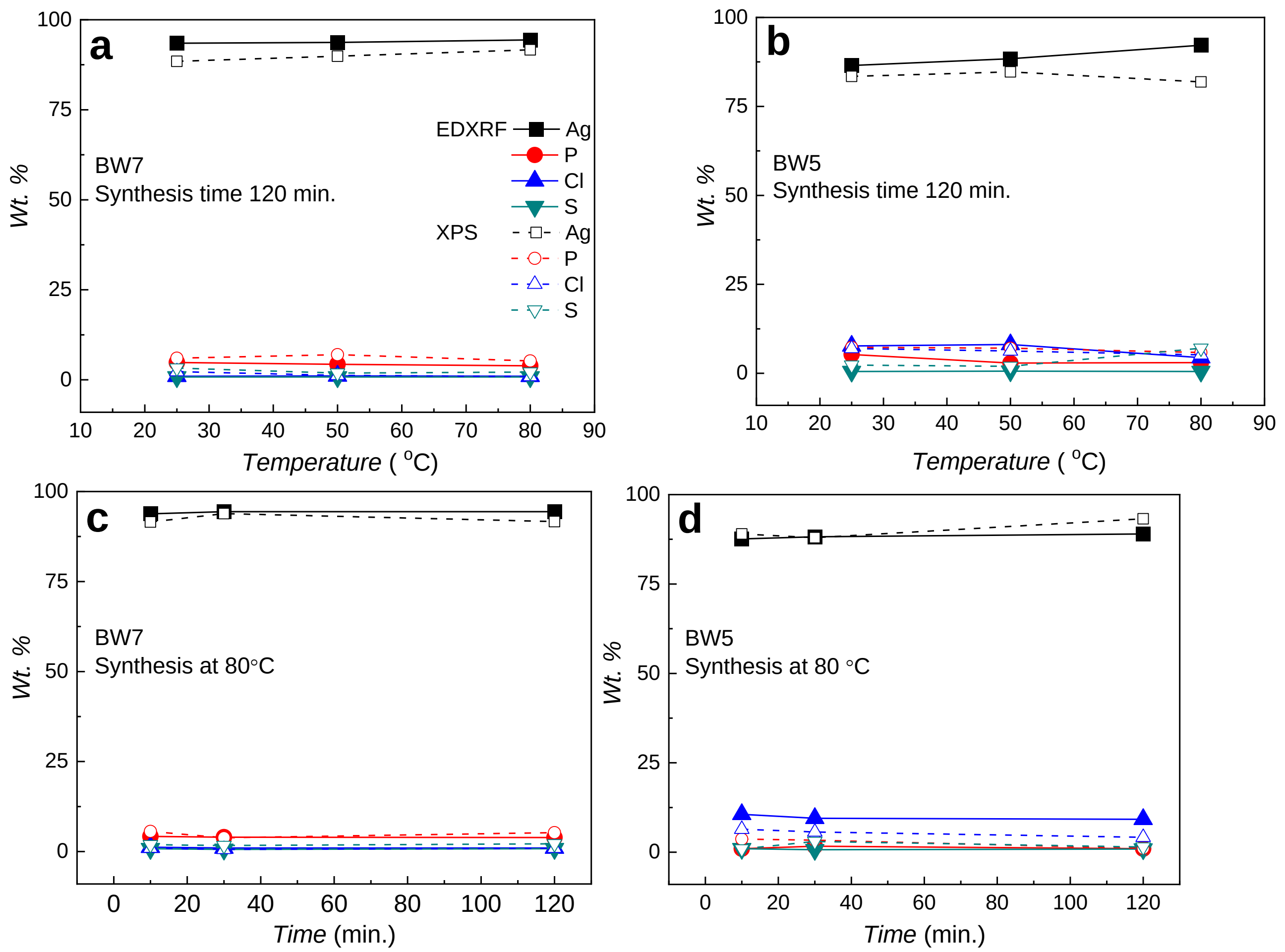

Figure S4. Comparison of elementary weight composition resulting from EDXRF and XPS spectra of nanomaterials synthesized at different temperatures and times using brewery wastes (a, c) BW7 and (b, d) BW5.

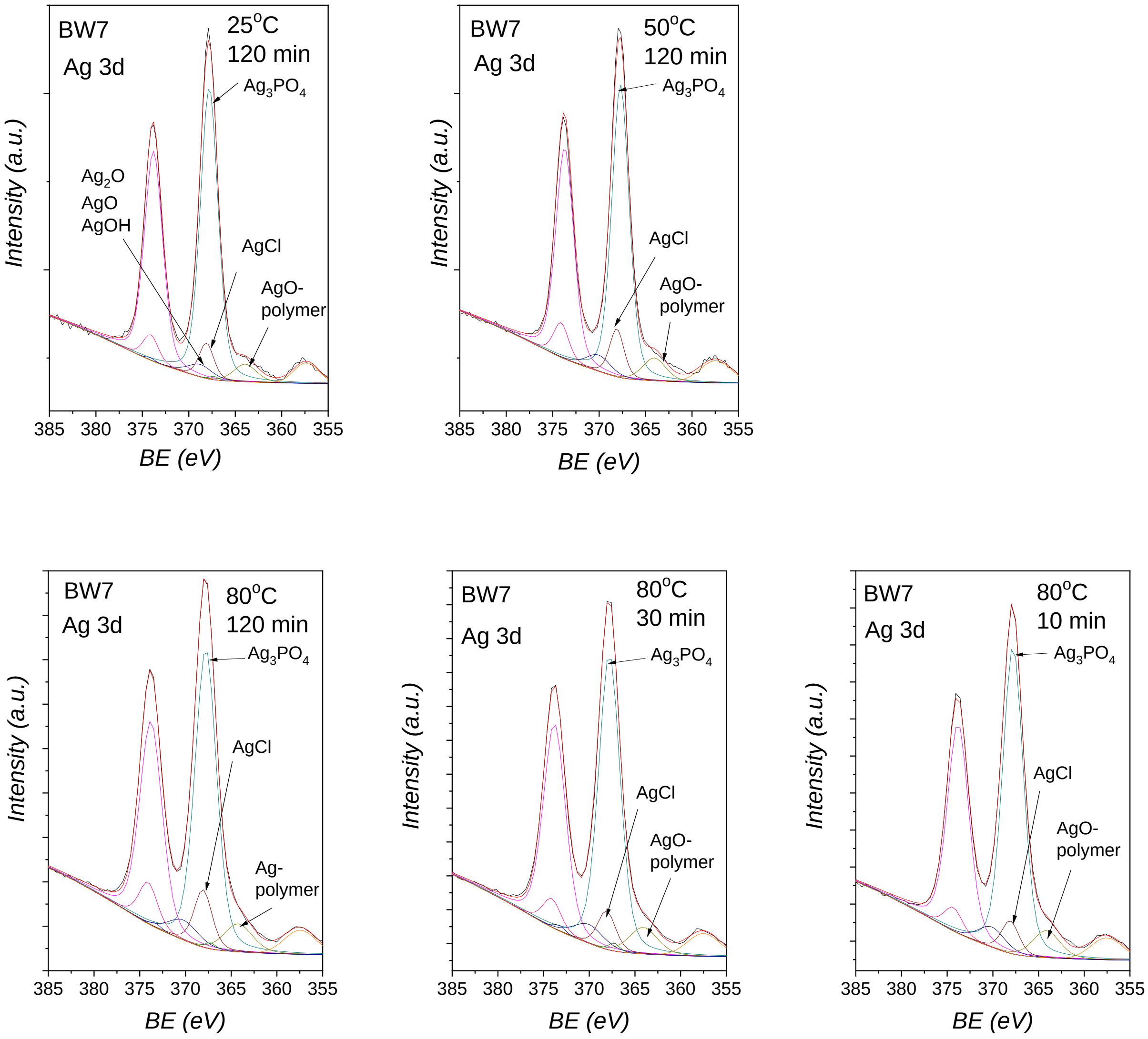
BW7
Ag 3d
25°C
120 min
Ag3PO4
Ag2O
AgO
AgOH
AgCl
AgO-
polymer
Intensity (a.u.)
385 380 375 370 365 360 355
BE (eV)
BW7
Ag 3d
50°C
120 min
Ag3PO4
AgCl
AgO-
polymer
Intensity (a.u.)
385 380 375 370 365 360 355
BE (eV)
BW7
Ag 3d
80°C
120 min
Ag3PO4
AgCl
Ag-
polymer
Intensity (a.u.)
385 380 375 370 365 360 355
BE (eV)
BW7
Ag 3d
80°C
30 min
Ag3PO4
AgCl
AgO-
polymer
Intensity (a.u.)
385 380 375 370 365 360 355
BE (eV)
BW7
Ag 3d
80°C
10 min
Ag3PO4
AgCl
AgO-
polymer
Intensity (a.u.)
385 380 375 370 365 360 355
BE (eV)

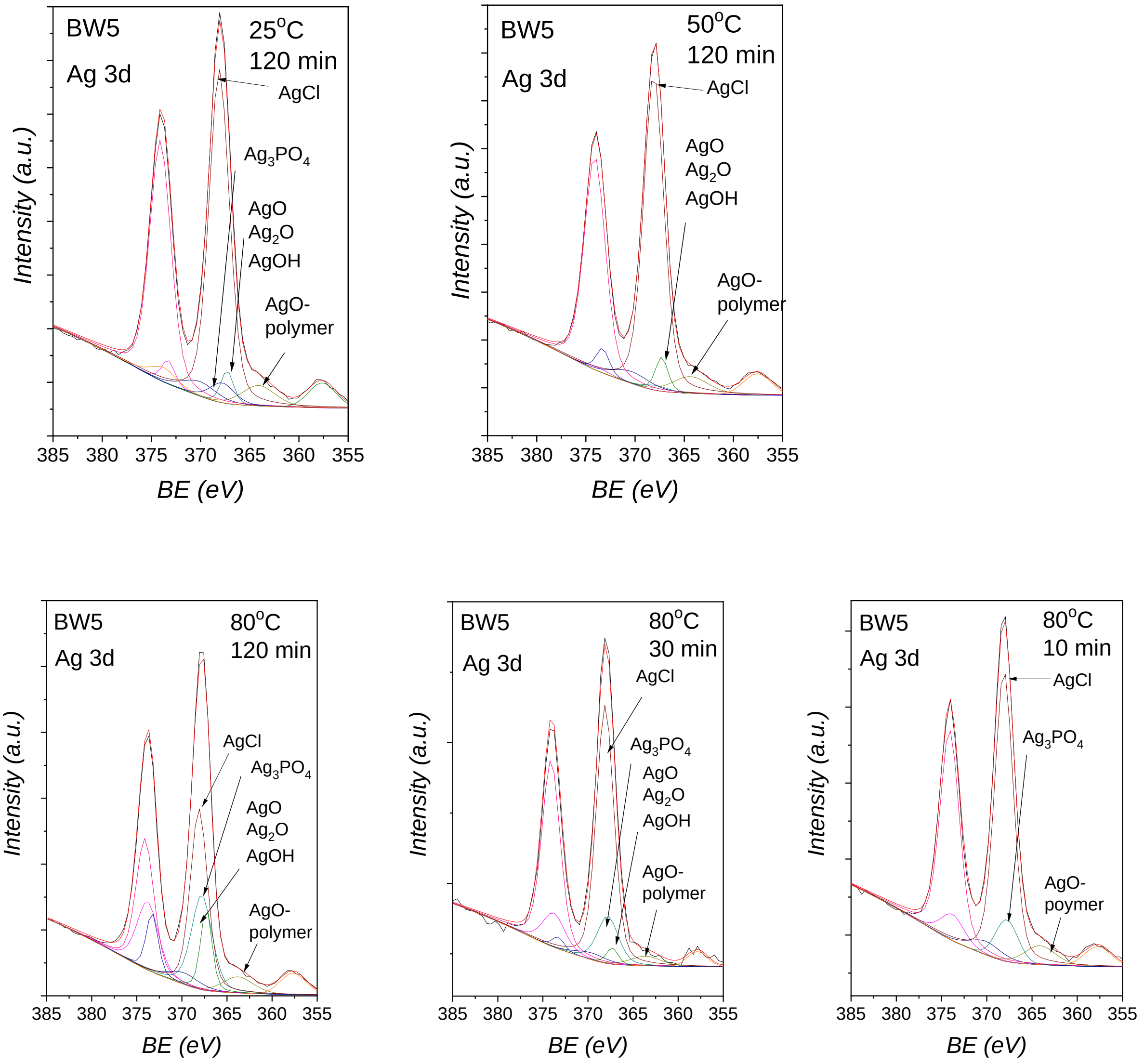

Figure S5a. The XPS Ag $3d_{5/2-3/2}$ spectra recorded from BW7 and BW5 nanomaterials synthesized at different temperature and time conditions fitted using Gaussian-Lorentzian asymmetric functions to different atomic chemical states.

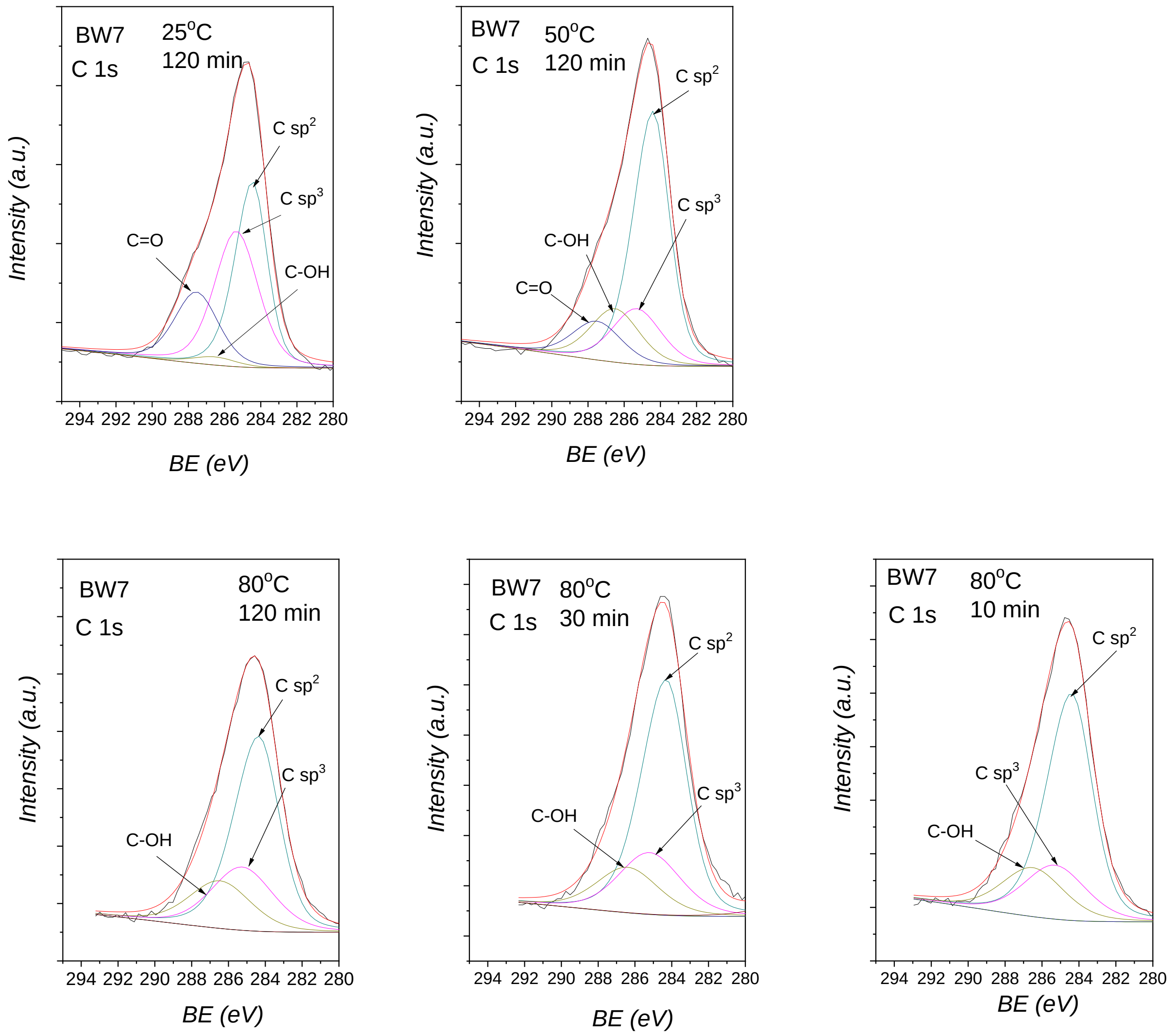
BW7
C 1s
25oC
120 min
C sp2
C sp3
C=O
C-OH
Intensity (a.u.)
BE (eV)
294 292 290 288 286 284 282 280
BW7
C 1s
50oC
120 min
C sp2
C sp3
C-OH
C=O
Intensity (a.u.)
BE (eV)
294 292 290 288 286 284 282 280
BW7
C 1s
80oC
120 min
C sp2
C sp3
C-OH
Intensity (a.u.)
BE (eV)
294 292 290 288 286 284 282 280
BW7
C 1s
80oC
30 min
C sp2
C sp3
C-OH
Intensity (a.u.)
BE (eV)
294 292 290 288 286 284 282 280
BW7
C 1s
80oC
10 min
C sp2
C sp3
C-OH
Intensity (a.u.)
BE (eV)
294 292 290 288 286 284 282 280

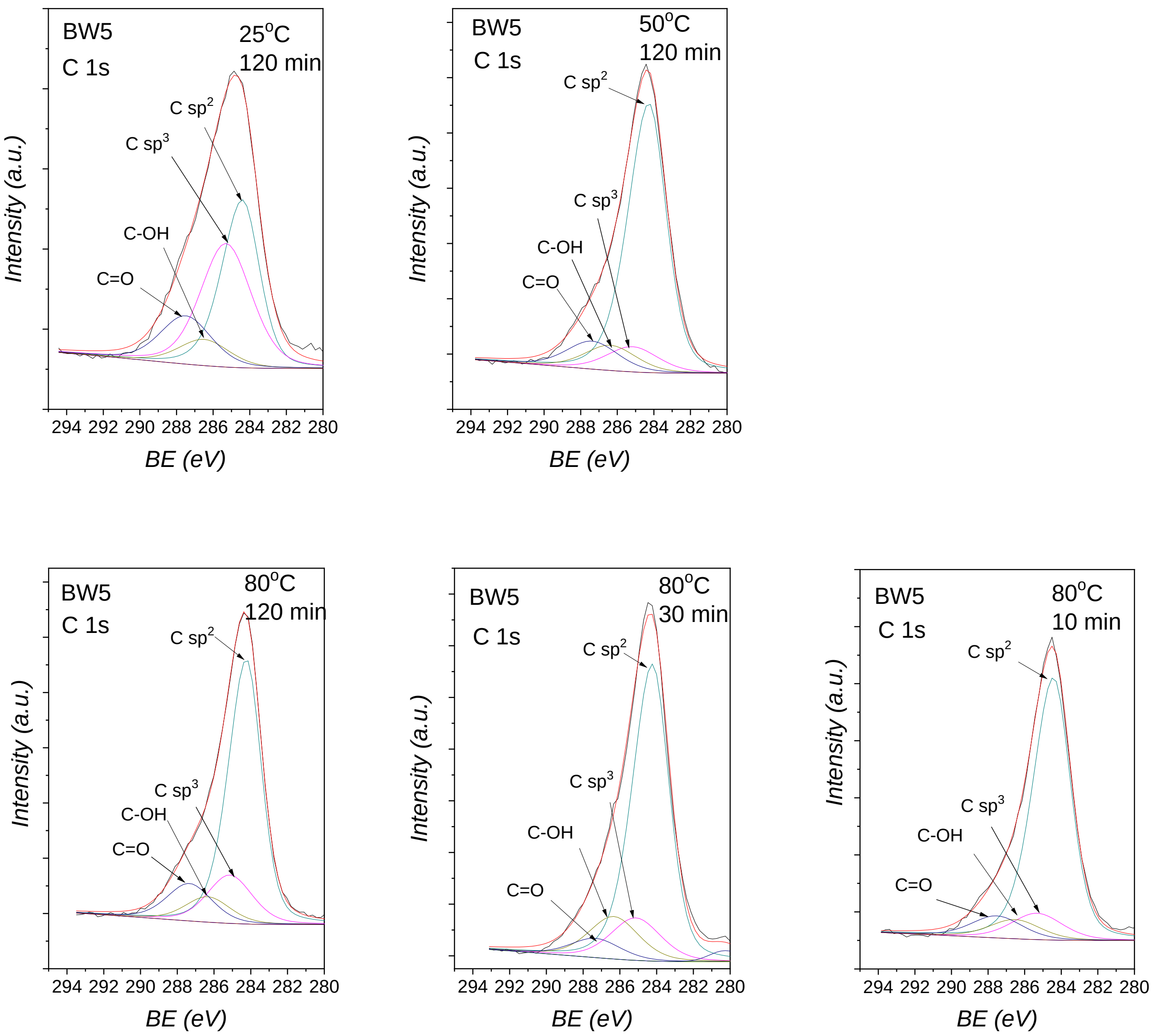

Figure S5b. The XPS C 1s spectra recorded from BW7 and BW5 nanomaterials synthesized at different temperature and time conditions fitted using Gaussian-Lorentzian asymmetric functions to different atomic chemical states.

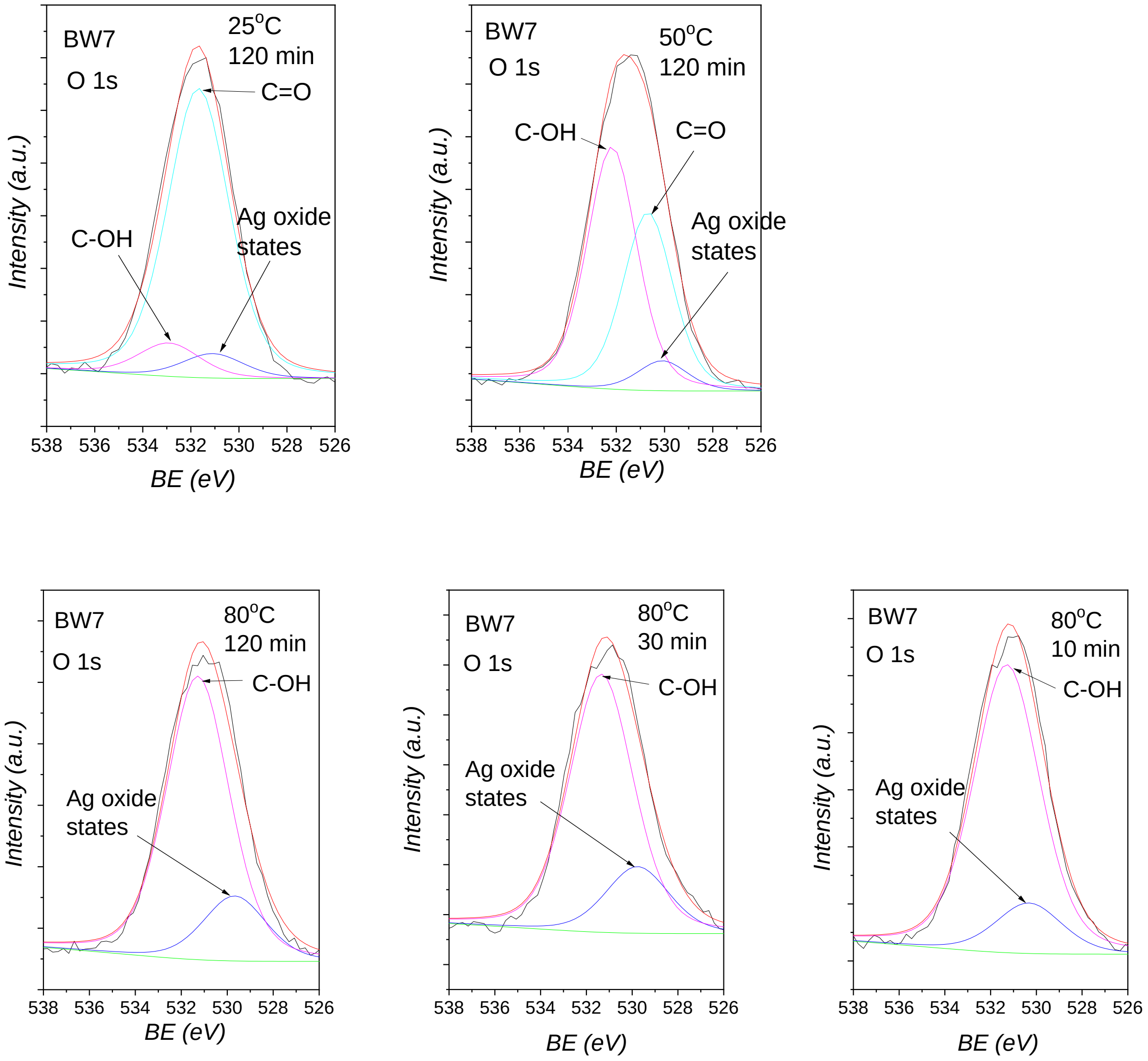
BW7
O 1s
25^oC
120 min
C=O
C-OH
Ag oxide
states
Intensity (a.u.)
BE (eV)
BW7
O 1s
50^oC
120 min
C-OH
C=O
Ag oxide
states
Intensity (a.u.)
BE (eV)
BW7
O 1s
80^oC
120 min
C-OH
Ag oxide
states
Intensity (a.u.)
BE (eV)
BW7
O 1s
80^oC
30 min
C-OH
Ag oxide
states
Intensity (a.u.)
BE (eV)
BW7
O 1s
80^oC
10 min
C-OH
Ag oxide
states
Intensity (a.u.)
BE (eV)
538 536 534 532 530 528 526

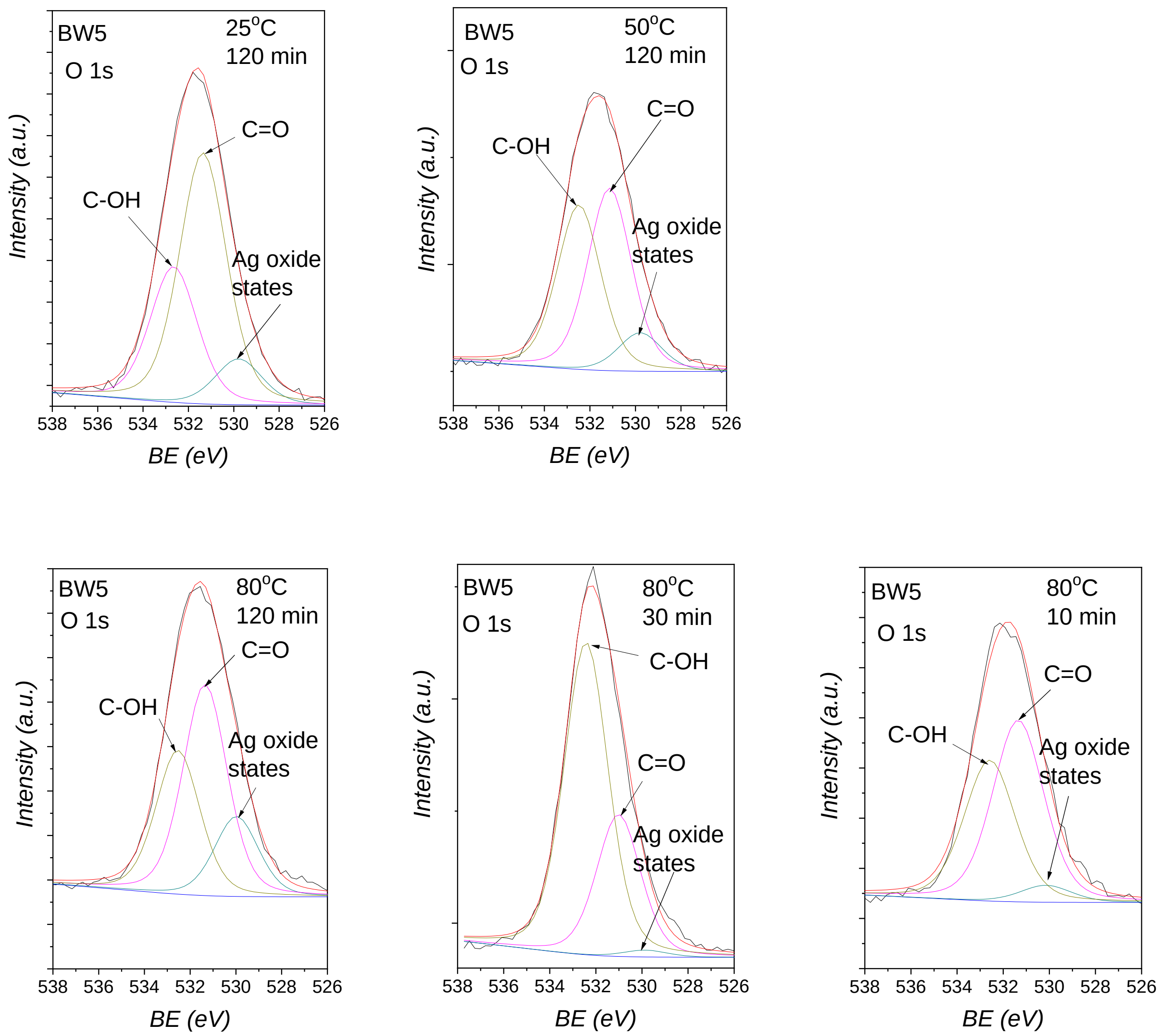

Figure S5c. The XPS O 1s spectra recorded from BW7 and BW5 nanomaterials synthesized at different temperature and time conditions fitted using Gaussian-Lorentzian asymmetric functions to different atomic chemical states.

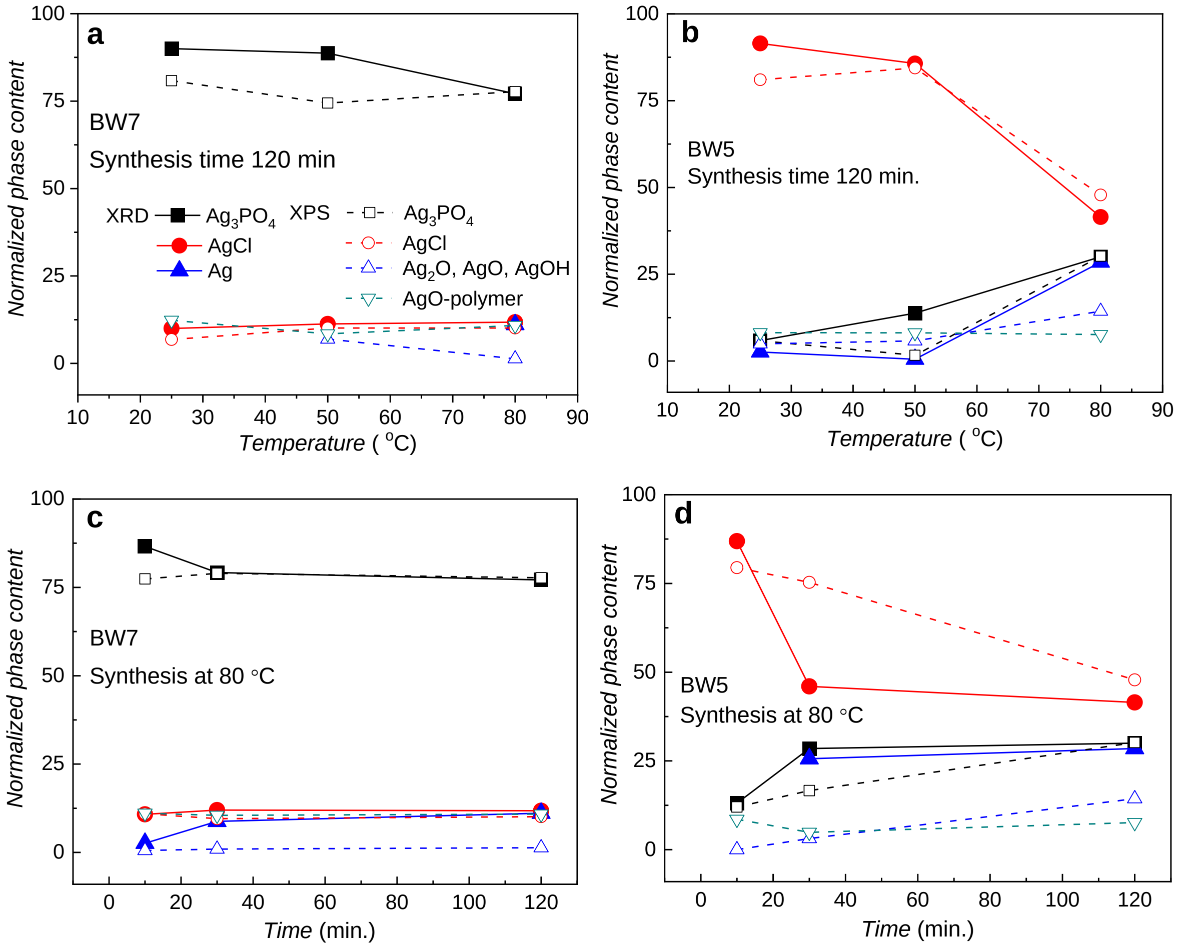

Figure S6. Comparison of weight and normalized phase content resulting from XRD and XPS spectra, respectively, in nanomaterials synthesized at different temperatures and time using brewery wastes (a, c) BW7 and (b, d) BW5.

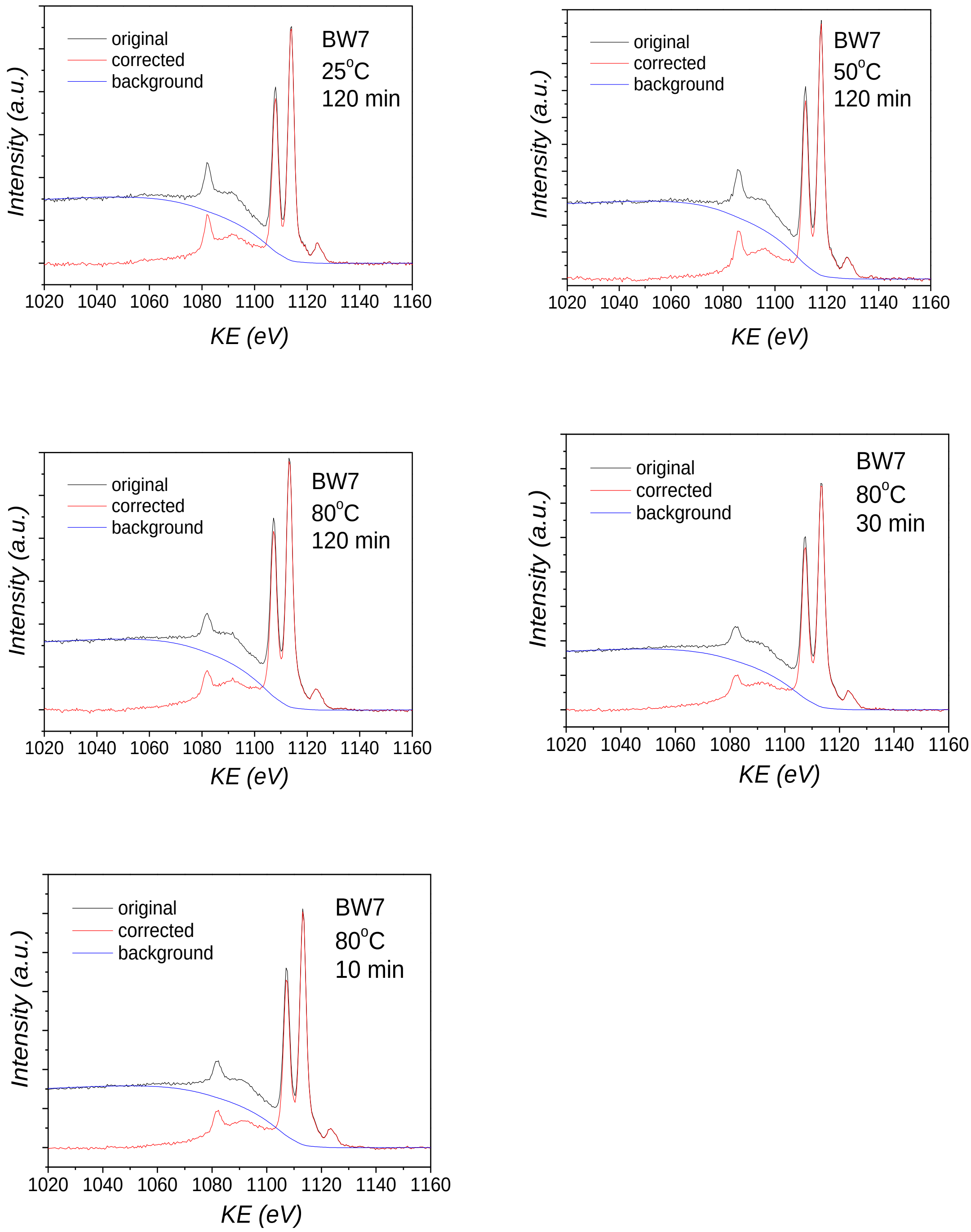

Figure S7a. Results of Ag $3d_{5/2/3-2}$ spectra analysis using QUASES-Analyze software and Buried Layer (BL) model for BW7 nanomaterials at different synthesis temperatures and times at 80 °C.

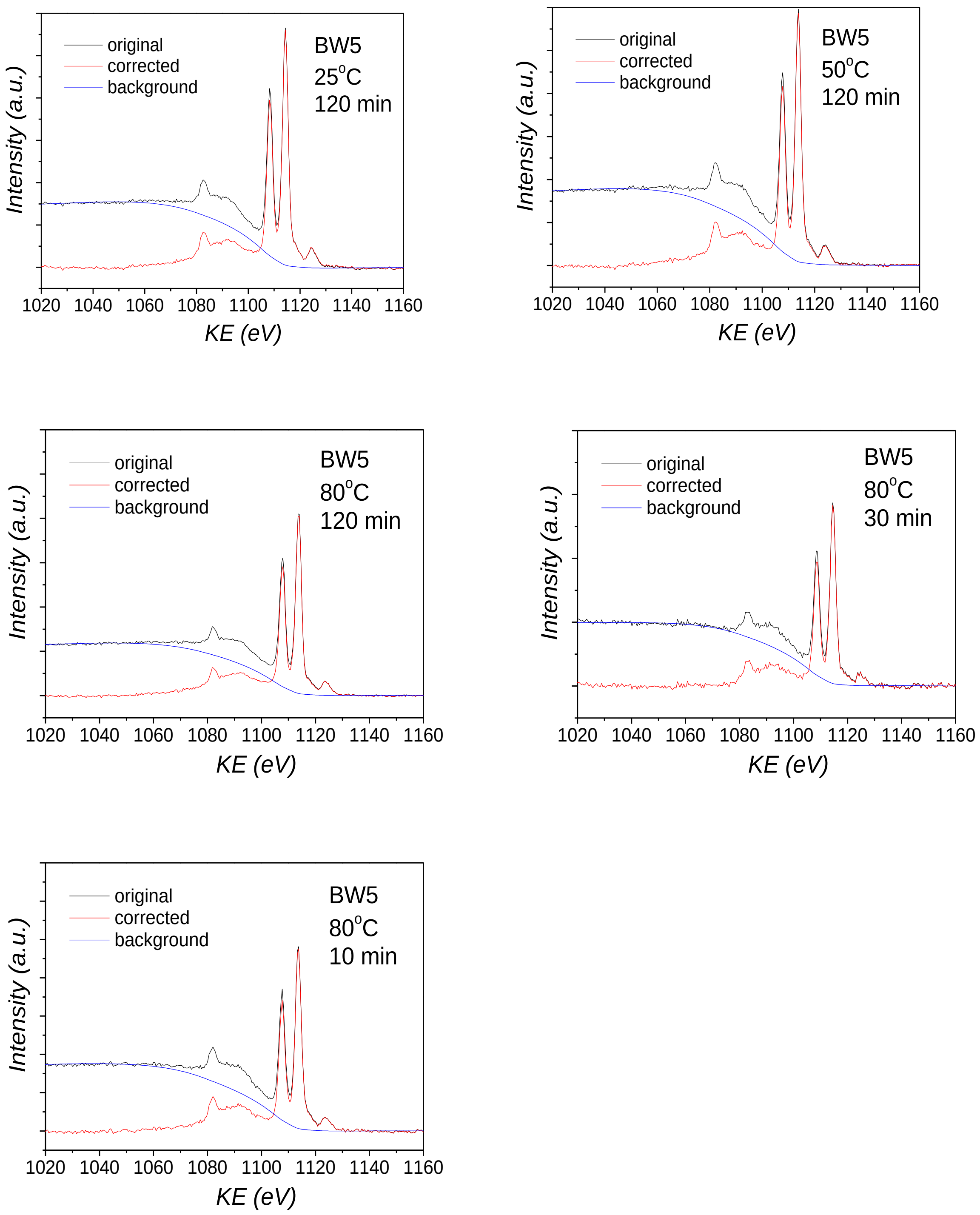

Figure S7b. Results of Ag $3d_{5/2/3-2}$ spectra analysis using QUASES-Analyze software and Buried Layer (BL) model for BW5 nanomaterials at different synthesis temperatures and times at 80 °C.
